# Hybrid-integrated devices for mimicking malignant brain tumors (“tumor-on-a-chip”) for *in vitro* development of targeted drug delivery and personalized therapy approaches

**DOI:** 10.3389/fmed.2024.1452298

**Published:** 2024-11-19

**Authors:** Tatiana M. Zimina, Nikita O. Sitkov, Kamil G. Gareev, Natalia V. Mikhailova, Stephanie E. Combs, Maxim A. Shevtsov

**Affiliations:** ^1^Department of Micro and Nanoelectronics, St. Petersburg Electrotechnical University “LETI” (ETU), Saint Petersburg, Russia; ^2^Personalized Medicine Centre, Almazov National Medical Research Centre, Saint Petersburg, Russia; ^3^Klinikum Rechts der Isar, Technical University of Munich, Munich, Germany

**Keywords:** brain tumor, tumor-on-a-chip, blood-brain barrier, organ-on-a-chip, microfluidic devices

## Abstract

Acute and requiring attention problem of oncotheranostics is a necessity for the urgent development of operative and precise diagnostics methods, followed by efficient therapy, to significantly reduce disability and mortality of citizens. A perspective way to achieve efficient personalized treatment is to use methods for operative evaluation of the individual drug load, properties of specific tumors and the effectiveness of selected therapy, and other actual features of pathology. Among the vast diversity of tumor types—brain tumors are the most invasive and malignant in humans with poor survival after diagnosis. Among brain tumors glioblastoma shows exceptionally high mortality. More studies are urgently needed to understand the risk factors and improve therapy approaches. One of the actively developing approaches is the tumor-on-a-chip (ToC) concept. This review examines the achievements of recent years in the field of ToC system developments. The basics of microfluidic chips technologies are considered in the context of their applications in solving oncological problems. Then the basic principles of tumors cultivation are considered to evaluate the main challengers in implementation of microfluidic devices, for growing cell cultures and possibilities of their treatment and observation. The main achievements in the culture types diversity approaches and their advantages are being analyzed. The modeling of angiogenesis and blood-brain barrier (BBB) on a chip, being a principally important elements of the life system, were considered in detail. The most interesting examples and achievements in the field of tumor-on-a-chip developments have been presented.

## Introduction

Cancer continues to occupy a leading position in disease-associated mortality, which is inferior only to cardiovascular diseases (1). The World Cancer Research Fund (WCRF) urges to prevent millions of deaths each year by raising awareness and education about cancer, and pressing governments and individuals across the world to take an active role in the fight against the disease (1). There exists a large variety of cancer types, among which brain cancers have proven to be the most fatal and responsible for substantial morbidity and mortality (2). They are invasive and malignant in humans with poor survival after diagnosis (3). Brain tumors include more than 120 different types, ranging from the least malignant, characterized by not invasive growth, such as meningioma (4), or common in children—medulloblastoma (5), and on the other hand, the most malignant, giving only months of survival for patients and showing extremely high mortality—glioblastoma (6).

Thus, at present time cancer, and brain tumors in particular, still significantly affect the life quality of patients, and it is necessary to admit that current treatment tactics achieve only limited effect (7). The new experimental, *ex vivo*, models are being extensively developed to deepen understanding of tumor growth, as well as to develop new versions of treatment since *in vivo* models demand high costs and labor resources while at the same time are considered ethically unacceptable by society (7). They also often fail to mimic crucial features of tumors. It is getting obvious that more studies are urgently needed to understand the risk factors and improve therapy approaches. It is also stressed by WHO, that many of these deaths can be avoided, since many types of tumors can potentially be detected early, treated, and cured (8). But at present time, screening methods for malignant brain tumors, unlike breast cancer or lung cancer, have not yet been developed, so there are still few opportunities for their early diagnosis.

Brain tumors are complex and heterogeneous structures consisting of various types of cells, such as tumoral, neuronal, glial, endothelial, microglial, astrocytic, oligodendroglia, etc. (9–11), which interact with each other and with the environment, forming the tumor microenvironment. The tumor microenvironment includes various factors, such as blood and lymphatic vessels, extracellular matrix, gradients of oxygen, glucose, pH, etc. (12–14). These factors influence tumor growth, metabolism, migration, invasion, angiogenesis, immune response, and resistance to therapy. To adequately model brain tumors *in vitro*, it is necessary to consider all these aspects and create organ-on-a-chip that maximally mimic the anatomy, physiology and pathophysiology of the brain and its tumor formations.

One of the promising areas of development (after the absolute priority area of screening and early diagnosis approaches) is the creation of means for studying dynamics, interactions in heterogeneous cellular media, therapy and many other aspects of growth and treatment of malignant tumors. This may be achieved by modeling of tumor tissues, particularly using new generation hybrid-integrated devices, such as organs-on-a-chip (OoC), which can be specifically designed for growing tumor cultures and observing their interactions with environment, including cells, anticancer medicines, liquids, etc. (15).

In general, OoC is a relatively new direction in modeling biological systems to study aspects of human pathophysiology and disease (16). OoC are miniaturized hybrid devices mimicking the structure and function of organs or organ parts *in vitro* that contain living cells and tissues. OoCs make it possible to reproduce structure and function of target fragments of human body, the processes that are developing in them, as well as to study the influence of various factors, such as drugs, toxins, stress conditions, infections, etc., on these processes. It appeared that multidisciplinary approach based on OoC shows quite a few advantages over traditional biological models of tumor research, such as single cells, spheroids, animal models, etc. For example:

- High level of authenticity, due to cultivation of living cells and tissues, which could be personalized, possibility to maintain as well as ability to maintain specified cultivation conditions, including temperature, pressure, pH, oxygen, nutrient media, mechanical forces, etc.
- High level of integration, achieved through the combination of different types of cells and tissues, as well as the inclusion of sensory and actuator elements, allowing to monitor and control the processes occurring in the OoC, as well as regulate various parameters of the microenvironment, such as fluid flow, pressure, temperature, concentration of oxygen, and nutrients.
- A high level of standard microtechnologies participation, allowing the use of a wide range of materials and the creation of functional units adaptively depending on the object being studied and the characteristics required for analysis.
- High level of personalization, achieved by the use of individual cells and tissues of patients, as well as adaptation of OoC parameters and conditions to specific needs and research goals.
- The opportunity of parallel testing of multiple samples and conditions on a single platform.

Current review analyzes the recent progress achieved in the field of, so-called, brain tumor-on-a-chip (ToC) developments. The basics of microfluidic chips technologies are considered in the context of their applications in solving oncological problems. Then the basic principles of tumors cultivation are considered to evaluate the main challengers in implementation of microfluidic devices, for growing cell cultures and possibilities of their treatment and observation. The main achievements in the culture diversity approaches and their advantages are being highlighted. The modeling of angiogenesis process and blood-brain barrier-on-a-chip, as principally important elements of the life system, have been considered in detail. The most interesting examples and achievements in the field of ToC developments have been considered.

## Fundamentals of manufacturing hybrid-integrated devices for mimiking brain tumor (tumor-on-a-chip): design, materials and technologies

The hybrid-integrated devices for mimicking malignant brain tumors, tumors-on-a-chip (ToC), considered in this review are used for modeling and studying brain tumors. In this section the design, materials and technologies of ToCs, as well as the biosensor elements integrated into them are considered.

### Tumor-on-a-chip topologies and designs

**Figure 1:**
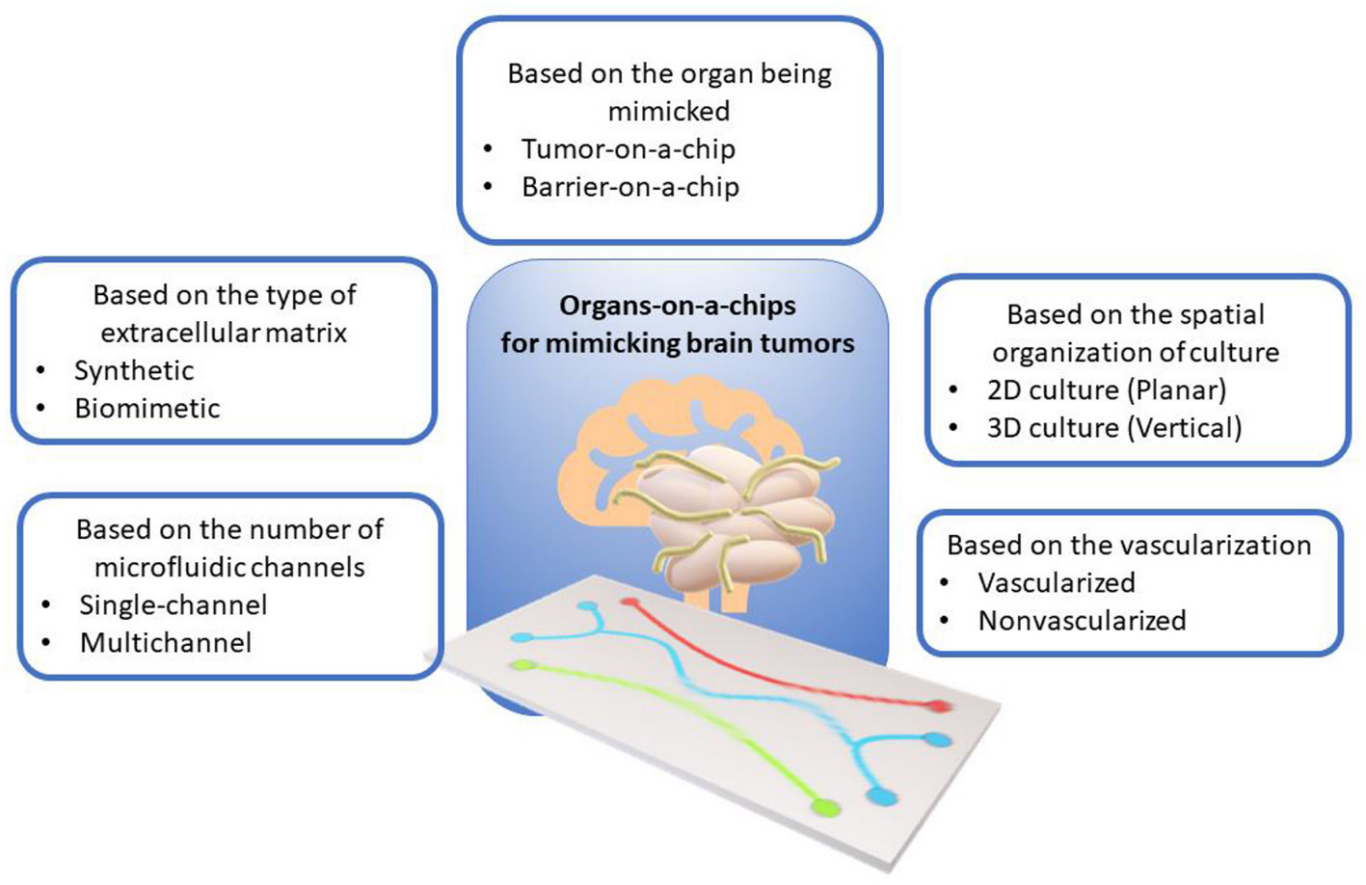
Classification of organ-on-a-chip models for mimicking brain tumors according to various criteria.

ToCs for modeling brain tumors can be classified according to various criteria, such as the type of cell structure being mimicked, the spatial organization of the culture, the number of microfluidic channels, the presence of vascularization, and the type of extracellular matrix. Figure 1 shows the classification of ToCs for mimicking brain tumors according to various criteria.

Depending on the type of structure being mimicked, the following main designs can be distinguished:

*Tumor-on-a-chip* (ToC) is modeling the microenvironment and structure of a brain tumor, including tumor cells, extracellular matrix, vessels, immune cells, and other components (25, 26). In relation to neuro-oncology, some authors may also use the term “brain-on-a-chip” (17, 18), but we believe that in this subject area it is more informative to use a term focused specifically on tumors. Such systems usually contain one or more channels filled with neuronal cells or tissues that can undergo electrical or chemical stimulation (19, 20). ToCs are convenient for studying growth mechanisms of tumors, including vascularization (21), invasion, metastasis, angiogenesis, immune response, and drug sensitivity (22–24, 27, 28). Tumor cells in Toc also could be exposed to gradients of oxygen, glucose, pH, mechanical forces, anticancer drugs, etc. (29–31).

*Barrier-on-a-chip* (BoC) models barriers formed between different tissues or organs, such as the blood-brain barrier (BBB) (32), blood-tumor barrier (blood -tumor barrier, BTB) (33), blood-cerebrospinal fluid barrier (BCSFB) (34), etc. BoCs make it possible to study the structure, function, permeability, transport, regulation, and disruption of barriers under normal and pathological conditions (35, 36). BoCs can also be used to screen and test drug candidates that must overcome barriers to reach the target tissues or organs (37). BoCs typically consist of two or more channels separated by a semipermeable membrane on which the different cell types that form the barrier are grown, such as endothelial, epithelial, astrocytes, pericytes, etc. (38–40). The use of epithelial cells in brain barrier models may seem incomplete or not entirely relevant from a physiological point of view, but there are several reasons for their use. Such cells are relatively easy to culture [e.g., MDCK (canine renal epithelial cells) or Caco-2 (human intestinal epithelial cells)] and they exhibit good barrier properties, making them suitable for basic research (41). The well-established culture protocols make them an attractive experimental target, especially in the initial stages of research. Epithelial cells, although not identical to BBB endothelial cells, can be used to assess basic barrier functions such as transport of substances across the cell layer, active and passive transport. In addition, primary BBB endothelial cells or specific brain cells may be difficult to obtain or expensive to obtain and culture. Epithelial cells, being more readily available, may be used as surrogate models in resource-limited conditions.

Depending on the type of culture, ToCs can be divided into the following categories:

- *ToC with 2D cultures*. These are the simplest and cheapest models in which cells are grown on a flat surface such as glass, plastic or polymer (42, 43). These planar models are easy to manipulate and analyze, but they do not reflect the realistic and complex structure and function of the tumor microenvironment.
- *ToC with 3D cultures*. These are more complex and expensive models in which cells are grown in a three-dimensional environment such as a hydrogel, matrix, or scaffold (44, 45). These models better mimic the complex structure and function of the tumor microenvironment, such as three-dimensional organization and function of cells, extracellular matrix, vascularization, barriers, gradients, forces, and other factors (46, 47). Based on three-dimensional cultures, it is possible to work with cell spheroids and organoids, which can consist of various types of cells (48). Such models are more realistic and informative. However, they are also more difficult to manipulate and analyze, and require high control over the microenvironment and integration with biosensors.

Based on the configuration of microfluidic channels, the following designs can be distinguished:

- *Single-channel ToCs* have a single microchannel in which tumor cells are directly examined (49) or can contain a culture chamber to maintain the necessary conditions (50). Single-channel ToCs allow mimicking a homogeneous environment and provide a constant flow of fluid and nutrients. This is important when studying the movement of anticancer drugs, especially those incorporated with magnetic nanocarriers. In addition, when trying to mathematically describe such complex systems, constant flow conditions allow us to form a more convenient approximation for calculation.
- *Multichannel ToCs* are those that consist of several channels connected to each other or to a common reservoir (51, 52). Such systems enable culturing of different types of cells and tissues to simulate more complex and realistic tumor microenvironments. Multichannel ToCs allow the simulation of heterogeneous environment and different regimes of fluid and nutrient flow to be provided.

Vascularization plays an important role in the development, growth, invasion, angiogenesis, and metastasis of brain tumors, as well as in the transport and drug toxicity testing. Depending on the presence of vascularization, the following main types of ToCs can be distinguished:

- *Non-vascularized ToCs* are those that do not have vasculature within or around cells or tissues. Non-vascularized ToCs allow to model avascular brain tumors or individual parts of them, as well as to study the effects of hypoxia, acidity, and metabolic stress on tumor cells (53–55).
- *Vascularized ToCs (VToCs)* are those that have vasculature within or around cells or tissues. Vascularized ToCs allow to model vascular brain tumors and study the influence of angiogenesis, perfusion, trans endothelial migration and drug delivery on tumor cells (56, 57). It has been shown that *VToCs* provide higher level of mimicry of the physiological conditions, particularly of blood and lymph circulation, which ensures the more authentic simulation and study of mechanisms of angiogenesis, metastasis, and drug delivery through the BBB (58).

Extracellular matrices (ECM), in the tumor microenvironment and, its influence on the behavior and live prospects of cells (59). The extracellular matrix consists of various proteins, polysaccharides, glycoproteins and glycosaminoglycans, which can form various structures, such as collagen fibers, fibronectin networks, basement membranes, etc. (60). The ECM can influence the morphology, proliferation, differentiation, migration, adhesion, invasion, and apoptosis of cells, as well as their signaling pathways and gene expression (60).

Depending on the type of ECM, the following main types can be distinguished:

- *Synthetic ECM* consists of synthetic materials such as polymers, gels, or membranes (61). Synthetic materials allow the control of the chemical composition, mechanical properties, and topography of the ECM, as well as ensuring its good biocompatibility and stability. However, synthetic ECM cannot reproduce the biological activity and heterogeneity of life tissues, nor does it reflect the physiological conditions of human ECM. Polyethylene glycol (PEG), polylactide-co-glycolide (PLGA), polydimethylsiloxane (PDMS), or alginate can be used as materials for creating synthetic ECMs. Such systems need to be modified with proteins, peptides, or other bioactive molecules to improve their ability to support cell adhesion and other functions important for cell cultures.
- *Biomimetic ECM* is composed of biological materials such as collagen, fibronectin, laminin, hyaluronic acid, or decellularized tissue (62, 63). Biomimetic ECM can reproduce the biological activity and heterogeneity of the natural tissues, and reflects the physiological conditions of the human ECM. However, biological ECM requires more complex preparation and sterilization techniques and has limited ECM controllability and variability.

Since ECMs are typically derived from natural sources (e.g., animal or human tissue), their sterilization is an important preparation step before use in biomedical applications. However, sterilization of ECMs is challenging because methods that effectively kill microorganisms (temperature, UV, radiation) can also damage or alter the structure and biological activity of the matrix. Therefore, an important challenge is to integrate this process into the overall workflow of ECM creation and to create effective sterilization protocols, taking into account the use of microfluidic systems.

Specific examples of various organ-on-a-chip designs and topologies for modeling brain tumors will be presented in the following sections.

### Materials for ToCs fabrication

The materials used to fabricate ToCs are selected according to their structure, characteristics and properties, such as:

- Biocompatibility—the ability of a material not to cause unwanted biological reactions such as inflammation, infection, and immune response or toxicity.
- Optical transparency is the ability of a material to transmit light, allowing cells or tissues within the OoC to be visualized and measured using optical techniques such as microscopy, spectroscopy, or fluorescence.
- Gas permeability—the ability of a material to allow gases, such as oxygen and carbon dioxide, to pass through, which allows to maintain the required level of gas exchange and metabolism of cells or tissues inside the OoC.
- Mechanical strength—the ability of a material to withstand mechanical loads, such as pressure, shear or tension, that may occur during the manufacture, storage or operation of the OoC.
- Chemical resistance—the ability of a material not to enter into chemical reactions with cells, tissues, fluids, or drugs that are inside or in contact with the ToC, thereby avoiding unwanted chemical changes or damage to the ToC.
- Thermal stability—the ability of a material not to change its properties or shape when the temperature changes, which may occur during the manufacture, storage, or operation of ToCs, which allows avoiding of unwanted thermal deformations or destruction.

Depending on the chemical composition, the following are the most common types of materials used for the manufacture of ToCs for mimicking brain tumors:

- *Elastomers* are polymeric materials with highly elastic properties—exhibit reversible extension with low hysteresis and minimal permanent set. The most widely used elastomer in forming microfluidics structures is polydimethylsiloxane (PDMS). It has the advantages of gas permeability, mechanical compatibility, chemical compatibility, low cost, and ease of processing (64). The main disadvantages of PDMS are hydrophobicity and low mechanical strength (65). In addition, the properties of PDMS may be degraded by thermal sterilization.
- *Thermoplastics* are materials that can be melted and molded at high temperatures and harden when the temperature is lowered. Thermoplastics such as polycarbonate (PC), polymethyl methacrylate (PMMA), polystyrene (PS), polyethylene (PE), polypropylene (PP), etc. are widely used for OoC fabrication due to their advantages such as high optical transparency, mechanical strength, chemical compatibility, low cost, and ease of processing (66, 67). However, thermoplastics have disadvantages, such as high thermal deformation, rather weak gas permeability, low electrical compatibility, possible autofluorescence and technological difficulties in integration with sensors and actuators (68, 69). In addition, joining and bonding thermoplastics to other materials or their own parts can be difficult. When creating thermoplastic chips, it may be technologically necessary to ensure sufficient hermeticity, especially under high pressure conditions or when long-term flow experiments are required. This can lead to leaks, poor compatibility with sealing materials, and problems with the durability of the device. Thermoplastics can also be coated with various materials such as silicone, polyethylene glycol (PEG), hydrogels, etc. to improve their biocompatibility, permeability, and antibacterial properties (70).
- *Glass* is an inorganic material that is formed when a molten mixture of silica and metal oxides is rapidly cooled. Glass is also widely used for the fabrication of OoC and ToC, as it has several advantages, such as high strength, sufficient biocompatibility, excellent optical transparency, good chemical stability, ease of microfabrication, and the ability to integrate sensors and actuators (71–73). Some of the most common types of glass that are used to make ToC are borosilicate glass, quartz glass, sapphire glass, pyrex, etc. However, glass has disadvantages such as poor gas permeability, low mechanical compatibility, high fragility, and possible difficulties in processing and heterogeneous integration with other materials.
- *Hydrogels* are materials that consist of networked polymer structures that can absorb and retain large amounts of water. Some of the most common types of hydrogels that are used to fabricate ToC are agarose, alginate, collagen, hyaluronic acid, gelatin, polyacrylamide, etc. (74–77). Hydrogels are quite often used for the fabrication of ECM in ToC due to their advantages such as high biocompatibility, mechanical compatibility, chemical compatibility, biological activity and variability. However, hydrogels have disadvantages such as low transparency, mechanical strength, thermal stability, and electrical compatibility.

**Figure 2:**
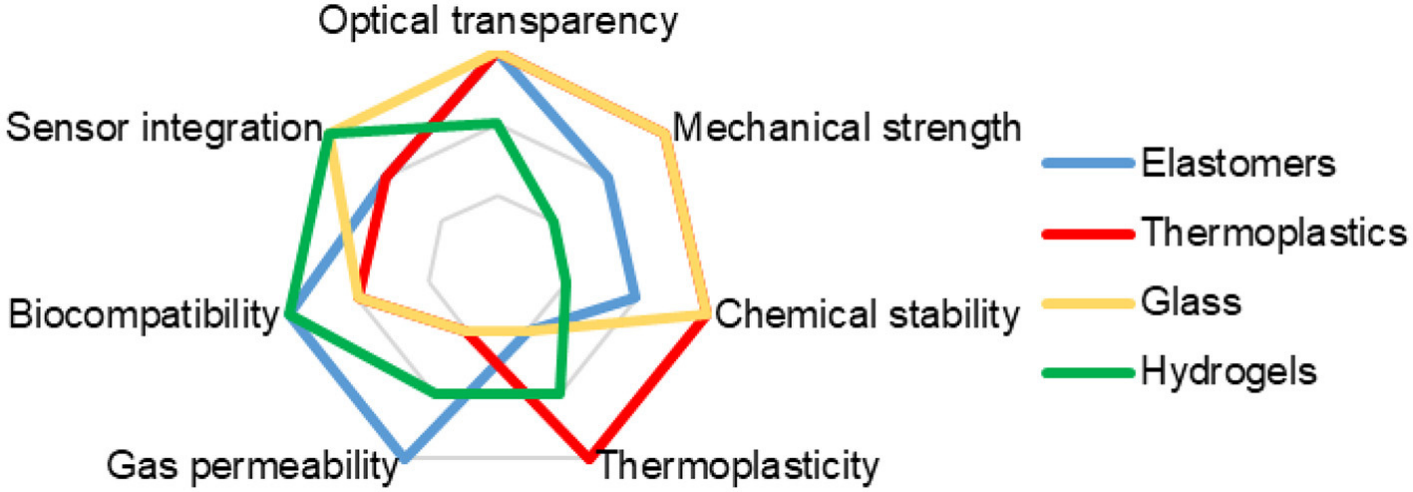
Comparison of the most common materials for the ToCs fabrication.

Figure 2 provides a comparison of the key properties of main materials used in the formation of ToC.

Some other materials as well as the most widely used once described earlier, can be useful in fabricating ToCs. These are: photopolymers (78, 79), silicon (80, 81) (including its use for creating master forms for soft lithography), metals for integrating sensor-actuator elements (82, 83), and membranes made of various materials (84, 85).

### Technologies for manufacturing ToCs

The technologies for brain tumor modeling must provide the ability to create microstructures with high precision, complexity and functionality, as well as the ability to integrate various materials, cells, tissues and sensor-actuator elements. Depending on the physical principle, the following are the most common types of technologies for mimicking brain tumors on ToC.

*Photolithography* is a technology that uses light waves to create micro- and nanostructures on the material coated with a photosensitive layer (photoresist) (86). Photolithography allows you to create ToC structures with high precision, speed, and flexibility, and is also compatible with various materials, such as thermoplastics, glass, silicon, etc. (87, 88). However, photolithography requires expensive and complex equipment, highly qualified personnel, and is also sensitive to contaminants and temperature.

*Soft lithography* is a technology that uses elastic templates, called masters, to create surface micro- and nanostructures in the layer called a replicant (89, 90). Soft lithography allows you to create ToCs with high accuracy, speed and flexibility, and is also compatible with various materials, such as PDMS, hydrogels, etc. The disadvantages of soft lithography may include the difficulty of controlling the structure and properties, as well as deformation and degradation of the template and material.

*Laser ablation* is a technology that uses laser beams to remove or modify material with high energy and precision (91). Laser technologies are characterized by high precision, flexibility, repeatability and a wide choice of materials. Laser ablation allows high precision manufacturing process due to the possibility of software control after automated design of the required structure (92). This group of technologies may have disadvantages such as increased channel surface roughness compared to other microfabrication methods (93), which can be solved through additional chemical surface treatment, and may also cause thermal damage or chemical changes to the material.

*3D printing* is a technology that uses digital models to create three-dimensional objects by depositing sequential layers of material (94). This group of technologies can use extrusion, injection and photopolymerization methods for the formation of microstructures (95). 3D printing allows you to create ToC of a more complex structure; it allows you to form using various materials not only microfluidic systems, reaction chambers, but also various auxiliary elements for the sustainable functioning of a hybrid device (96). However, 3D printing has rather low accuracy, speed, and mechanical strength. Fabrication of complex systems using this technology requires expensive and complex equipment.

*Bioprinting* is a technology that uses living cells and biomaterials to create three-dimensional tissues and organs by sequentially depositing layers of material (97, 98). Bioprinting makes it possible to ensure high biocompatibility and biological activity of manufactured structures, and is also compatible with various materials, such as hydrogels, collagen, alginate, etc. (99). Using this technology, it is possible to create complex geometries from living elements, simulate micro vessels and cultivate tumor cells in a vascularized system (100, 101). However, bioprinting has low speed and compatibility with integrated sensor-actuator elements, requires expensive and complex equipment, as well as strict control of cell sterility and viability.

To connect layers in microfluidic systems for preparation of ToCs, thermal bonding, connection with glue, photopolymerization, etc. are used (102).

**Figure 3:**
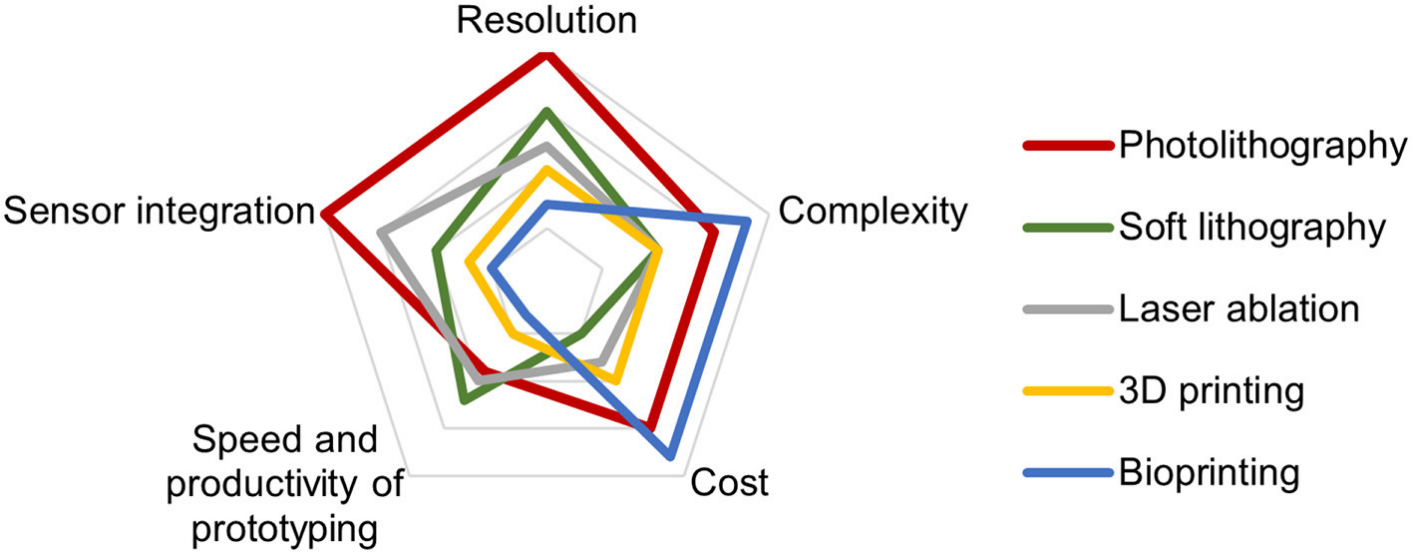
Comparison of common technologies for ToC fabrication.

Figure 3 provides a comparison of common technologies for ToC fabrication.

Thus, the diversity of materials and technologies allows researchers to create ToC systems of varying complexity to address a wide range of tasks. ToCs have become an important tool in studying the tumor microenvironment, invasion, angiogenesis, and drug response. Today, the key goal of creating such systems is to achieve the highest degree of biological similarity to ensure high experimental accuracy. Modern microtechnologies, such as photolithography, 3D printing, and bioprinting, enable the creation of complex microstructures and the integration of biological elements, although they sometimes require expensive equipment and high-tech processes. ToC systems are a promising tool for studying brain tumors. They allow for more accurate reproduction of physiological conditions, thereby increasing the efficiency of research into new therapeutic approaches and drugs.

## Specific features of growing cultures of brain tumor cells in microfluidic chips for mimicking tumors

Traditional two-dimensional (2D) brain tumor models cannot fully recapitulate the functionality and life stages of a brain tumor, nor do they reflect the physiological conditions of the human brain. Therefore, the development of more realistic and predictive models of brain tumors is an important task to improve the understanding of the functioning of brain tumors and the development of new therapeutic strategies.

**Figure 4:**
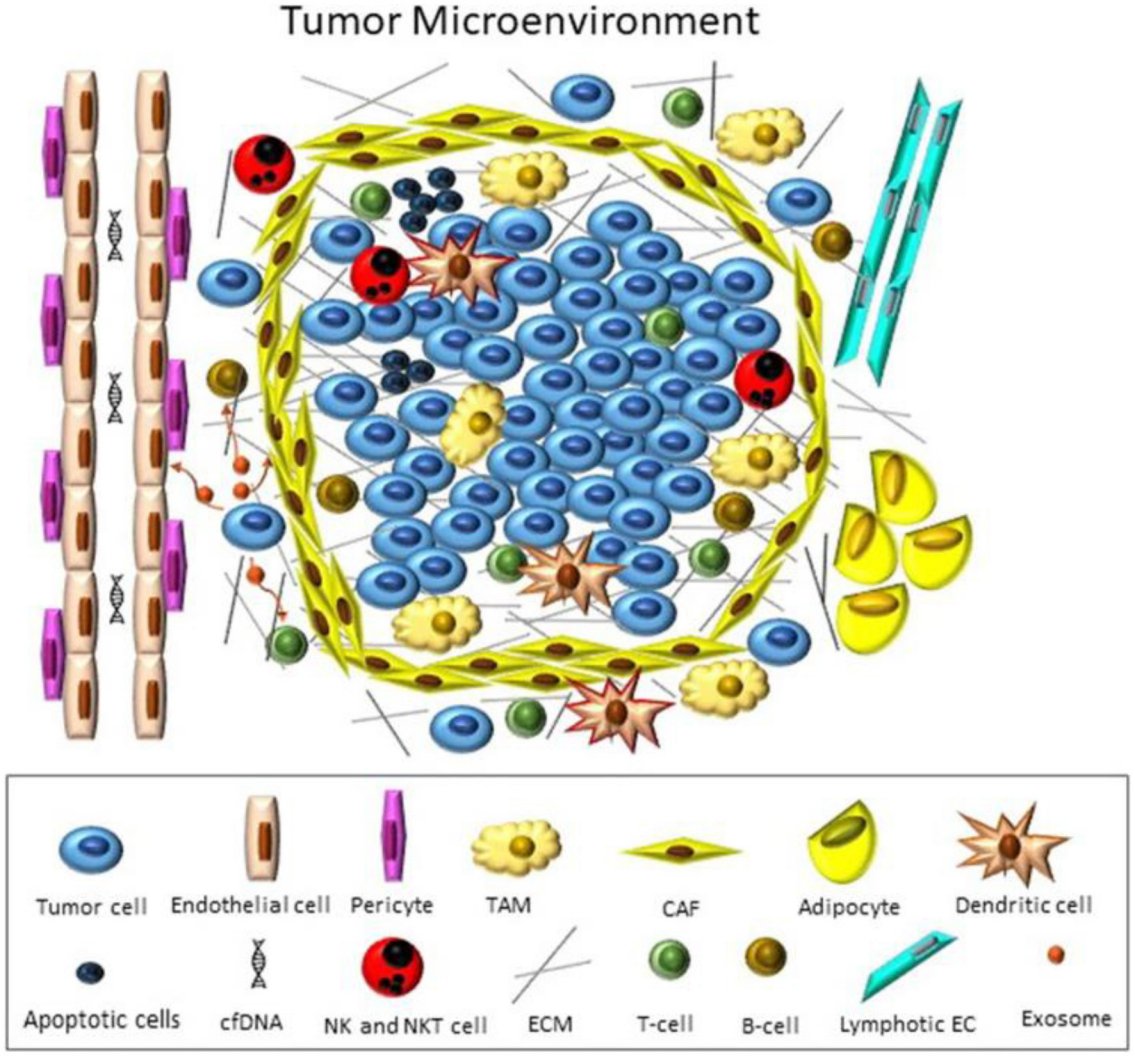
Tumor microenvironment. Tumor cells hijack different cellular and non-cellular non-malignant components of TME to promote their own growth and survival under hostile conditions. Meanwhile, the mediators for such contacts can be soluble factors (chemokines/cytokines/growth factors, etc.), or those that enable horizontal genetic/biomaterial transfer including cfDNA, apoptotic bodies, CTCs, and exosomes. Reprinted from Baghban et al. (106), license CC BY 4.0.

Growing cultures of brain tumors to improve understanding of tumor development and explore new therapies, is a labor intensive and multistage process, when exploring traditional classical approaches (103–105). In human body tumors develop under complex and dynamic conditions which influence their growth, invasion, and metastasis. In the life systems components are under communication and influence on each other. Such communications include cell-cell contacts as well as the mediators enabling these contacts. The mediators include molecules as extracellular vehicles (EVs), providing horizontal transfer of genetic information (Figure 4) (106).

Scaling of operations and integrating them into hybrid microfluidic devices—ToCs, may offer a new technical level of performing these tasks of tumor culturing and investigation. The implementation of this approach requires a detailed analysis of all stages of the process of culturing the malignant tumor cells. A variety of approaches used in this process have been discussed elsewhere (107). The traditional process of creating an organotypic culture is complex and multi-stage (108), and implementation of ToCs for culture growth makes the procedure less labor intensive and more accessible (109–111).

### Cultivation of tumor

**Figure 5:**
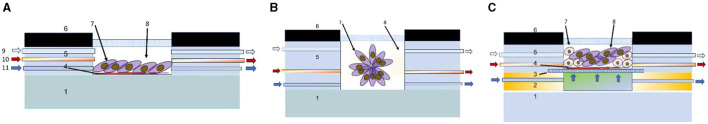
Schematic cross-sectional view of main tumor cultivation chambers integrated in microfluidic systems for cultivation of 2D culture **(A)**, spheroids **(B)**, and 3D heterogeneous co-culture **(C)**. 1—substrate (polymer, glass, silicon, etc.), 2—profile for nutrient flow arrangement, 3—porous support, 4—protein layer, 5—gas delivery channel, 6—casing, 7—tumor cells, 8—growth medium, 9—gas inlet, 10—pharmaceutical compounds inlet, and 11—liquid nutrient inlet.

Several cultivation techniques *in vitro* as noted earlier is applied for tumor modeling to test different therapeutic approaches (112) including 2D cell culture, 3D spheroids of various composition and morphology, 3D grown or printed heterogeneous co-culture (113), etc., which is schematically generalized in Figure 5.

The implementation of 2D culturing could be completed on a solid substrate, covered with special underlayers, such as collagen-based gels, Matrigel, etc. The 2D cell cultures could be seeded by injection, or by the capillary flow of suspended cells. The process is generally less labor intensive and expensive than 3D culturing. However, planar 2D cell cultures are well-known to have major limitations because the cancer cell behavior is restricted via the lack of biological and mechanical cues, they would naturally experience *in vivo*. Saydé et al. provide a comparison of 2D and 3D cultures (119). They note that when comparing the morphology and cell differentiation in 2D cultures, the cells lose their natural shape and polarization, while in 3D cultures they retain their real shape and polarization, which more accurately reflects their behavior in the body. In 3D cultures, spontaneous differentiation, and morphogenesis are observed through cell contacts or soluble factors, as well as a better representation of growth factors and genes associated with angiogenesis. In 2D cultures, these processes are modified and less physiological. 3D cultures allow the study of the functional heterogeneity of tumors and offer a more accurate representation of cell proliferation and response to drugs, while in 2D cultures this is limited to observation. However, 2D cultures are better at modeling the immune response, but 3D cultures provide better geometry and structure-function relationships, although complex co-cultures can complicate modeling. 2D cultures are more affordable, while 3D cultures, especially those using complex techniques, are more expensive.

Scaling down the most traditional 2D culturing onto a microfluidic chip needs preparation of the bio substrate at the bottom layer of microfluidic device cultivation chamber, which could be a solid material. However, cell viability may depend on the interactions with neighboring cells, as well as with the ECM (114), insufficient cell breathing due to the lack of vascularity, incorrect values of power and shear stress, which altogether or separately cause changes in cell phenotype during *in vitro* culturing (115–117), etc.

A variety of 3D culturing methods which can be applied for cultivation of spheroids includes anchorage-independent, or anchorage-dependent platforms (44, 118). Anchorage-dependent platforms provide cells with structural support and require attachment to a substrate for growth, which is important for tissue modeling and differentiation studies. Anchorage-independent platforms allow cells to grow without the need for attachment, making them useful for modeling tumor aggregation, metastasis, and other processes characteristic of cancer cells. Anchorage-dependent platforms could be in due course classified into hydrogel or scaffold-based types, porosity, density, and mechanical strength (119). Such technologies are usually applied for preparation of 3D spheroids, including basic models of tumors and multicellular tumor spheroids. The tumor spheroids are formed as solid spherical 3D cultures formed via proliferation of a single tumor stem/progenitor cell which are capable of self-organization after penetration into sphere-like particles (120, 121). Classification of basic principles for formation of anchorage-independent and anchorage-dependent cultures presented in Wanigasekara et al. (44).

Growing cells under serum-free, non-adherent conditions enables the enrichment of the cancer stem/progenitor cell population to be achieved since only this type of cells can survive and proliferate in this environment (121). It has been shown that 3-D culture methods are able to preserve the natural characteristics of tumors much better than conventional 2-D monolayer cultures, which applies to tumor-derived organoids, tumor-derived spheroids, organotypic multicellular spheroids, and multicellular tumor spheroids (122). Spheroids are unique in that they contain cancer stem cells (CSCs). They have been used as surrogate systems, derived as floating spheres, to evaluate the CSC-related characteristics of solid tumors *in vitro*, and have been established from numerous cancer types including glioma (122). They are applied as a high-throughput screening platform or for the cultivation of CSC-related tumor cells (122). A three-dimensional (3D) multicellular tumor spheroid to quantify chemotherapeutic and nanoparticle penetration properties *in vitro* have been described in Ma et al. (123). It is important to mention that multicellular tumor spheroids (MCTS) are able to capture some aspects of the dimensionality, cell-cell contact, and cell-matrix interactions seen *in vivo* which makes them attractive tool for tissue modeling. Many approaches exist to create MCTS from cell lines, and they have been used to study tumor cell invasion, growth, and how cells respond to drugs in physiologically relevant 3D microenvironments (124). However, there is often a problem of size and shape uniformity in generating tumor organoids. Lee et al. facilitated *in vitro* uptake of the NPs into the tight 3D tumor spheroids by the semi-spherical shape of the NPs with a proper size and surface charge (125).

An important problem in modeling cancer tissues is that the process is time consuming and have limitations, such as a lack of reproducibility and a wide distribution of spheroid sizes. Therefore, many researchers have used engineering methods, such as microfluidic devices, to culture 3D cells in dynamic conditions. Thus, a simple and low-cost microfluidic device fabricated from polymethyl methacrylate (PMMA) was proposed for dynamic and static cell culture examinations and *in vitro* monitoring of 3D cells growing. The liposome-based nanocarriers were used as the drug to test cell viability in 3D (126, 127). The approach is expected to improve *in vitro* testing models, reduce, and eliminate unsuitable compounds, and select more accurate combinations for *in vivo* testing (126–128).

At the same time, despite organoid techniques attractive features, such as possibility of multichannel testing of drugs and viability of tumor cells, comparative cooperativeness, there are still several disadvantages, and first the specific morphology of spheroids, which is not always perfectly consistent with the native tumor tissue and its environment. That is why the tendency of development tumor-on-a-chip systems for 3D tissue growing under special conditions more closely resembling the life tissues. Such new approaches enable the architecture of cells to be preserved in an extent allowing prediction of toxicity and antitumor drug resistance. Another achievement in 3D tissue engineering is the development of tools for mimicking the tumor microenvironment, enabling the evaluation of metastatic progression and vascularization. However, the costs and reproducibility problems are preventing the vast implementation of such approaches (129, 130). While standardized 3D culturing procedures could both reduce data variability and enhance biological relevance (129–133).

Micro methods could considerably improve the performance and quality of the testing using tumor cultures of various structures. Thus, microwell arrays can provide a facile method to produce uniform-sized spheroids in a high-throughput manner (134). Hydrogel microwell arrays were fabricated using a PDMS stamp as previously described (135, 136).

It has been demonstrated that the method of generating uniform-sized multicellular 3D tumor spheroids using PDMS microwell arrays could be a powerful tool for *in vitro* drug screening applications. Thus, the co-culture spheroids may have a homogeneous size of < 5% standard deviation, which is comparable to mono-culture spheroids of the same size (137). Such uniform-sized multicellular tumor spheroids are helpful in studying the effect of anticancer drug-loaded nanoparticles on their size or viability. In addition, the homogeneous and uniform-sized 3D tumor spheroids generated in the hydrogel microwells show tight cell-cell junction compared to heterogeneous models.

In creating organoid structures of tumors, it is perspective to use more advanced approaches of organogenesis, such as the one presented for the stem cells growing into organoids. This process requires the biochemical stimulus from exogenous signals, also called morphogens, such as proto-oncogene protein (WNT3), and Noggin (138). Brain organoids are valuable models for the study of human brain diseases and researchers are now in need of improved culturing and imaging tools to capture the *in vitro* dynamics of development processes in the brain. Khan et al. described the design of a microfluidic chip and bioreactor, to enable the *in situ* tracking and imaging of brain organoids on-chip under morphogenesis process (139). The low-cost 3D printed microfluidic bioreactor supports organoid growth and provides an optimal imaging chamber for live-organoid imaging, with drug delivery support. The fully isolated design of a live-cell imaging and culturing platform enables long-term live-imaging of the intact live brain organoids as it grows and to analyze thus their self-organization in a controlled environment with high temporal and spatial resolution. Also, spheroids- and organoids-on-a-chip technology could control the spatial distribution of the morphogens, magnifying the response and revealing their functions, using various strategies, such as gradient-concentration model, etc. (139).

**Figure 6:**
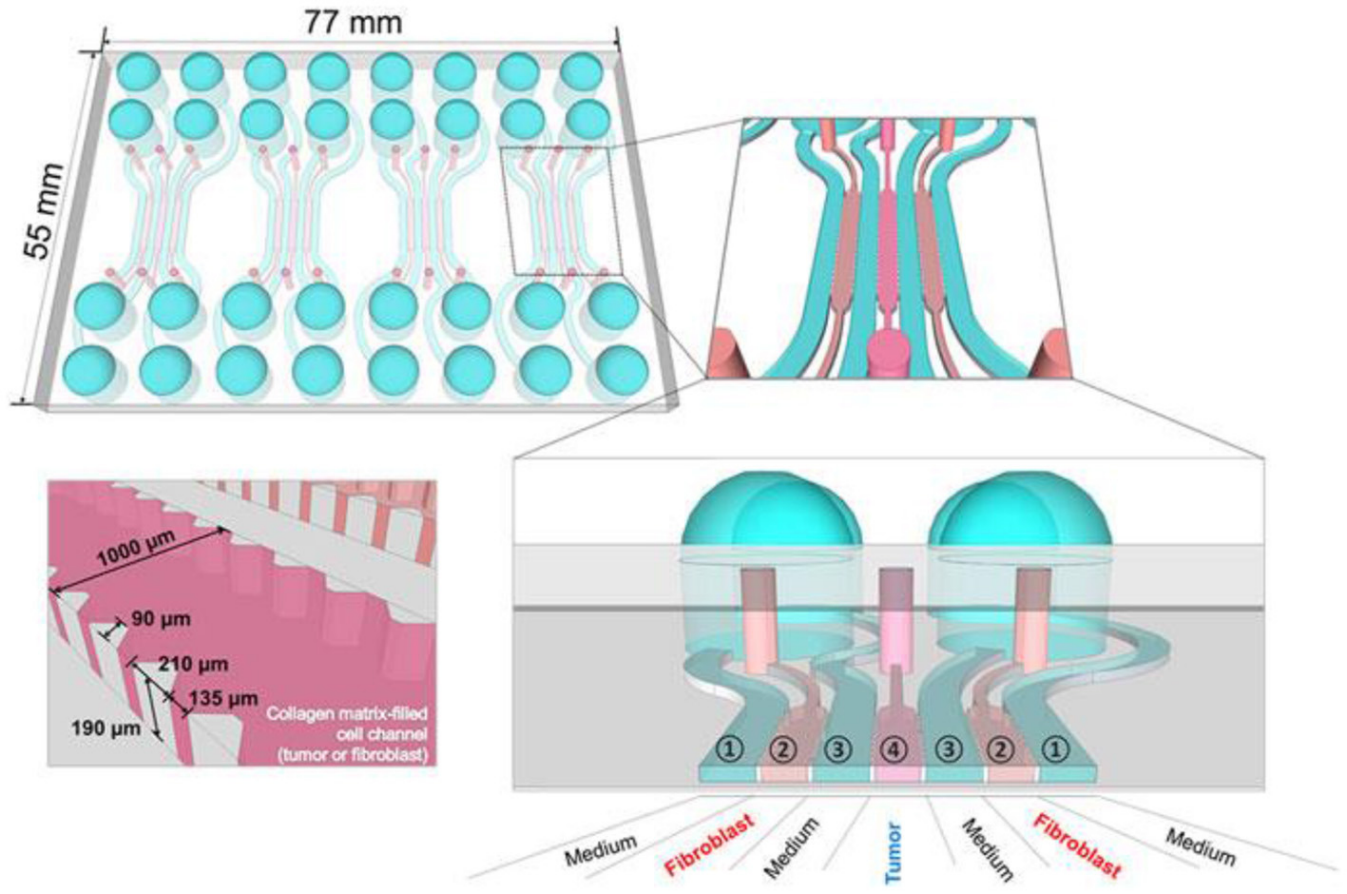
ToC consists of channels that can be loaded with media and cells and can support perfusion culture. Reprinted from Jeong et al. (142), license CC BY 4.0.

Further developments in organoids engineering technologies have enabled to overcome the limitations in reconstituting the perfusable microvascular system of large-scale tumors conserving their key functional features, via the reconstruction of micro vascularized organoid models using technologies of microfluidic systems (Figure 6).

Existing 3D tumor models have enabled *in vitro* simulation of the tumor environment (140–143), and some progress has been made in vascularization using different models (144). Shoval et al. prepared spheroids by mixing cancer cells of different origins with endothelial cells in different ratios, hoping to obtain 3D tumor models with vascular networks. Authors demonstrated the formation of capillary-like structures which were formed upon assembly and growth of mixed spheroids and that spheroids' shape and surface texture may be an indication of spatial invasiveness of cells in the extra-cellular matrix. Biogenic solutions for vascularization of tumor cultures will be considered in the next section.

Although spheroids are considered an ideal model mimicking some important tumor features, such as structural organization and the gradients of oxygen, pH, and nutrients (145), several issues with applying this model at a preclinical level remain, particularly reproducibility and high-throughput application (146). Recently, 3D bioprinting has been recognized as a promising technology for creating a tissue-based platform with high reproducibility and scalability (147–149). Obviously, a key feature in developing a 3D bioprint model useful in pharmaceutical applications in oncology is the choice of an appropriate printable biomaterial, commonly referred to as bioink, essential for determining cancer cell phenotypes and biophysical properties of the tissue. One of the main advantages of proposed 3D *in vitro* model is that it offers over the conventional 2D culture plates the ability to model and quantify cell invasion in a 3D cell culture setting (150). One advantage of the described design is its ability to create tumoroids in a compartmentalized array of microcavities that are connected to a microchannel (151, 152).

Thus, the 3D tissue cultures represent an experimental model able to potentially mimic *in vivo* growth more closely. They show distinct characteristics in terms of cellular phenotype, mass transport, and cell–cell interactions as compared with conventional 2D cell cultures. At the same time among 3D cultures organoids and printed tissues have certain limitations, namely some models of well-matrices generate spheroids of a wide distribution of sizes, due to variations inside the same well (153–155). At the same time vascularization, while being a critical value for tumor development and drug delivery, is still missing in 3D models (156, 157). While large-scale investigations and high-throughput tests are expensive and time consuming (158), and variability in biological matrices can lead to unpredicted experimental results (159–162).

**Figure 7:**
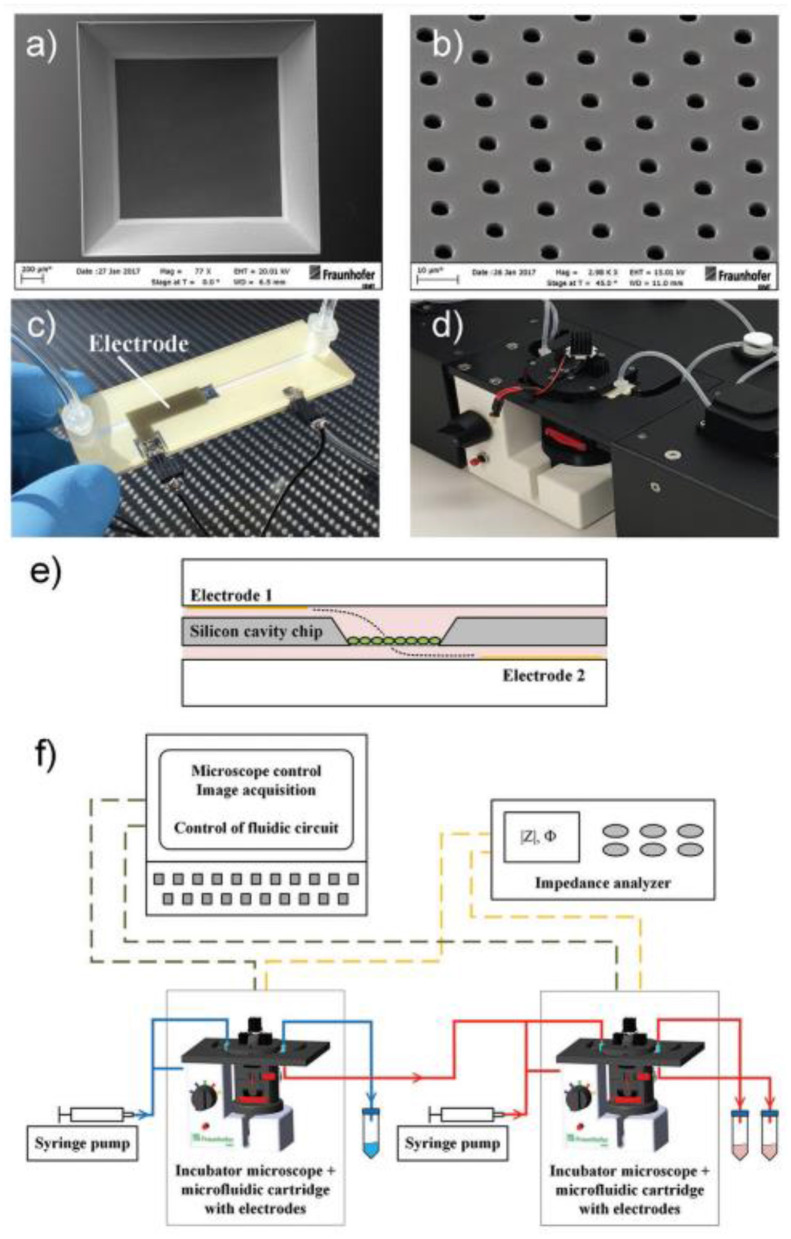
Setup of microfluidic cartridge and miniaturized incubator microscope platform. **(a)** SEM image of microcavity with micro hole array membrane (1.6 mm x 1.6 mm). **(b)** SEM image of micro hole array with hole distance 10 μm. **(c)** Microfluidic cartridge with connected tubing and cables for impedance measurement. This cartridge is integrated in the miniaturized incubator microscope. **(d)** Incubator microscope with inserted microfluidic cartridge and lid, for bright field imaging and cell cultivation under controlled temperature. **(e)** Schematic illustration of the microfluidic cartridge. The electrodes (yellow) are positioned in the two fluidic channels. The electric current flows between the electrodes through the pores in the membrane (dashed line). **(f)** Schematics of experimental setup with two platform modules for parallel or serial operation. Reprinted from Kohl et al. (164), license CC BY 4.0.

More appropriate are the heterogeneous 3D models comprised environmental cells and tumor cells (163). For preparation of this type of cultures, it is suitable to use hybrid microstructures, enabling the growth conditions to be provided. In Figure 12 the universal microfluidic device for cell culturing is presented. When preparing a conventional both 2D or 3D culture it is important to provide an appropriate substrate surface. In case of 2D cultures it could be a macroporous polymeric layer, impregnated with the nutrient, while in the case of 3D culture growth it is important to enlarge the specific surface area of the interface between the surface of the cell structures and the liquid nutrient, gas medium, etc. A universal solution could be the porous substrate (such as nanoporous anodic alumina) layer delivering the nutrient from the lower chamber (Figure 7) (164). At the same time such microsystem could be equipped with autonomous life supporting system.

The volume with nutrient has inlet and outlet openings with valves and ferrules, enabling a control over the content of the nutrient. The top chamber contains two or more inlet/outlet openings, enabling the delivery of cells, pharmaceuticals, markers, etc. On the outer circle of the top surface of the chamber, the inlets for air delivery are installed. The camera is covered with a glass window for monitoring of the culture state. The design enables various types of cultures to be grown, including the heterogeneous 3D cultures, containing TME.

**Figure 8:**
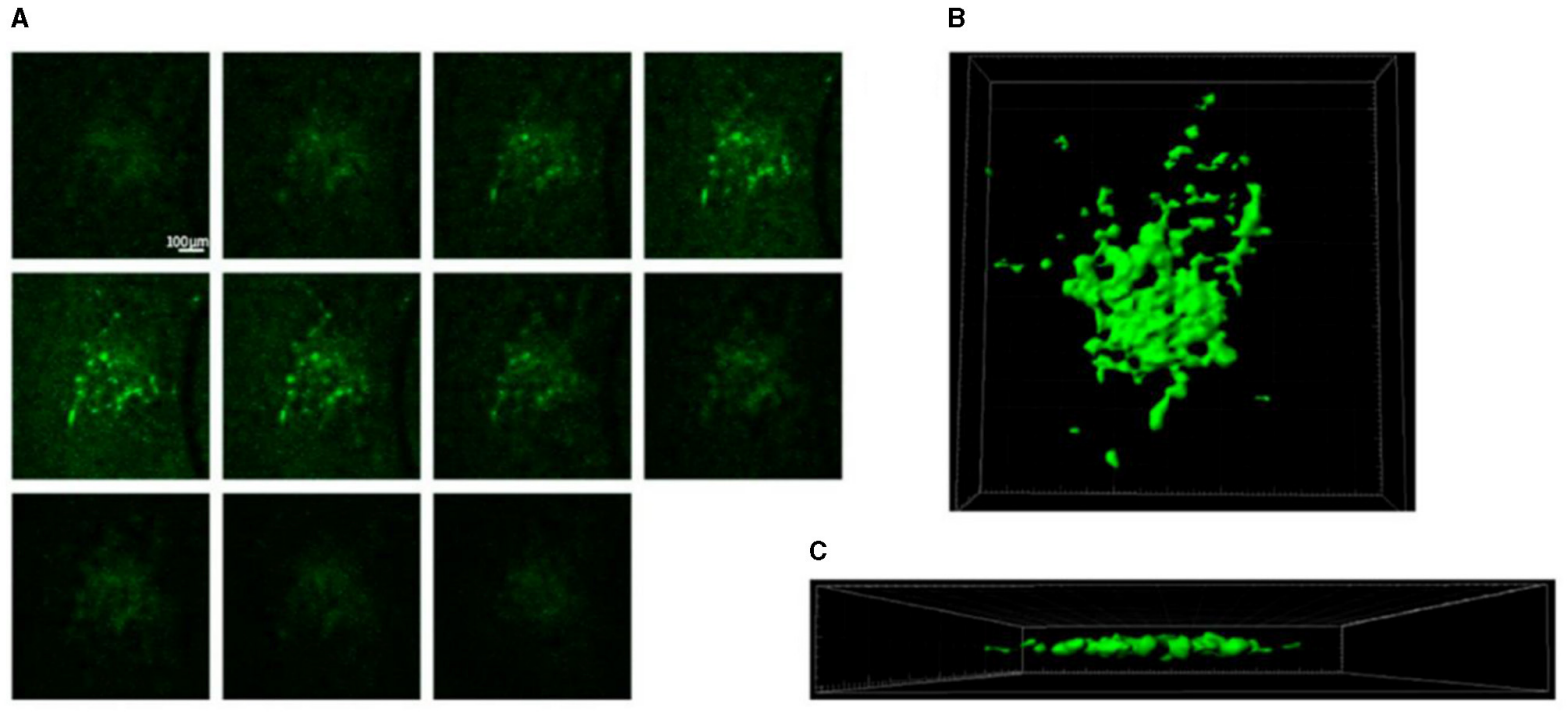
Samples sliced and seeded in the microfluidic device; **(A, B)** top view. **(C)** Side view. The bar is 100 μm. Reprinted from Chong et al. (108), license CC BY 4.0.

Chong et al. described the intact brain samples sliced and seeded in the microfluidic device (Figure 8), thus demonstrating that tumor slices provide a broadly useful platform for studying the tumor microenvironment and evaluating the preclinical efficacy of cancer therapeutics (108).

Robertson-Tessi et al. developed a hybrid multiscale computer model of tumor growth within a normal, homeostatic tissue, and demonstrated the mechanisms by which phenotypic, temporal, and spatial heterogeneity affect growth of a tumor and the outcomes of treatments (165). This work highlighted the importance of the phenotypic heterogeneity of tumor cells. A key prediction of this model is that early stages of tumor development (either primary or metastatic) maintain a phenotypically spatially structured population, where less aggressive clones spatially suppress more aggressive counterparts and that standard treatment modalities may selectively destroy this structured population and facilitate subsequent progression. Controlling tumor progression by maintaining rather than destroying this suppressive tumor layer appears to be more effective that conventional high-dose density therapy that aims to kill the maximum possible number of tumor cells (165).

A promising approach to creating tumors-on-a-chip are organotypic models of brain tumors, which attempt to reproduce the complex architecture and microenvironment of brain tissue as accurately as possible. Such models are created from fragments of brain tissue (usually from animals or humans) and preserve natural cellular interactions, including connections between neurons, glial cells, and the extracellular matrix. For example, Sivakumar et al. demonstrated rapid tumor cell expansion and invasion into a brain tissue substrate over relatively short periods of time (166). The monitoring was carried out using GFP fluorescence demonstrated the appearance of aggressively invasive tumor cell characteristics of the SMA-560 (spontaneous murine astrocytoma) cell line (166). The technique is a perspective alternative to *in vivo* models of brain tumor growth applicable in ToC technologies.

A simple and less resource-intensive, high throughput platform for further tumors studies is presented by Rodriguez et al. (167). Authors prepared a digitally manufactured microfluidic device in a biocompatible thermoplastic material by laser-cutting and solvent bonding. The fluidic performance of the device is described and demonstrates efficient on-device drug-response testing with tumor slices (167). The reported microfluidic platform is intended for functional drug testing of live tumor.

**Figure 9:**
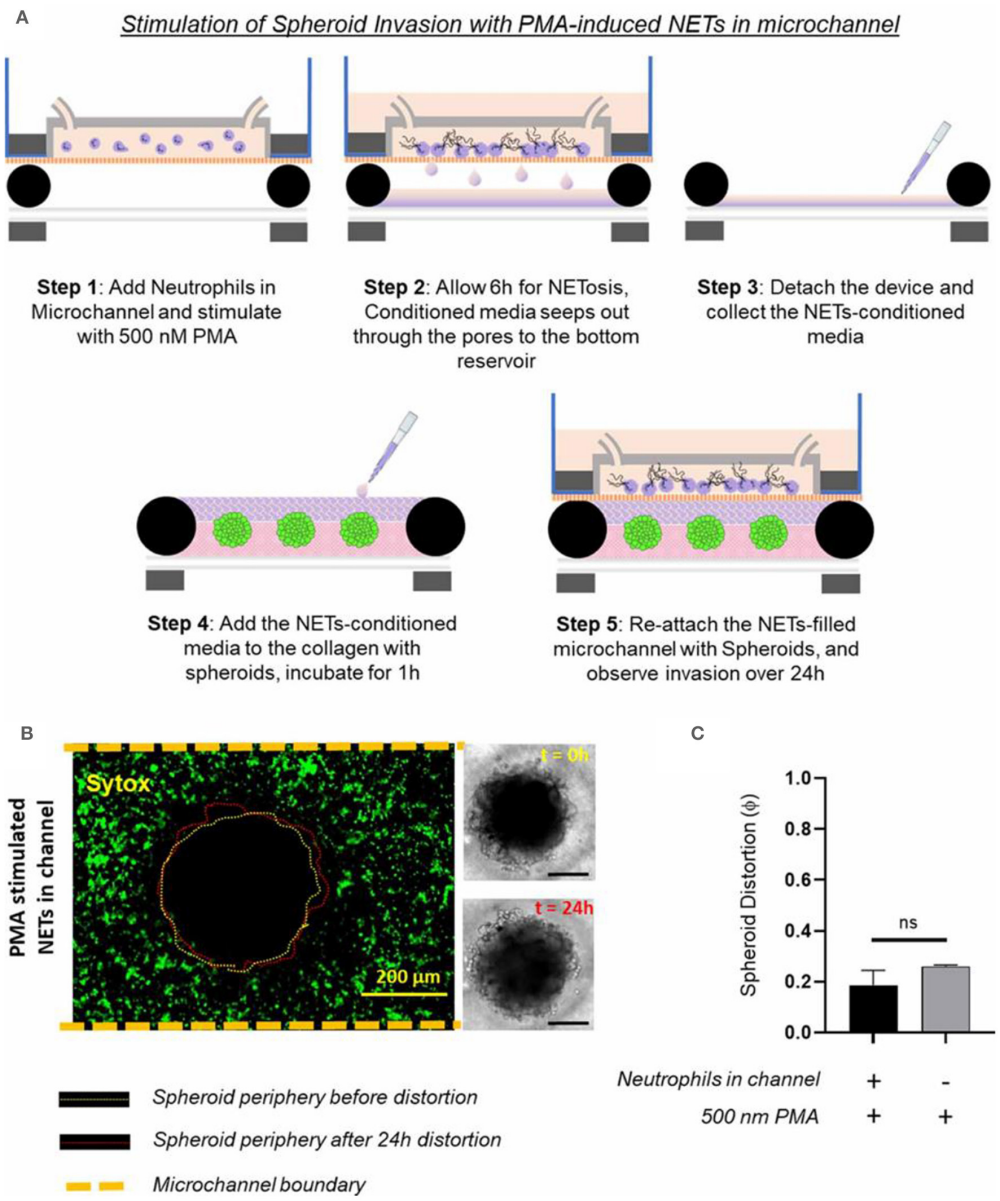
Testing of how NETs production outside the collagen region induces tumor invasion within a TIME, by separate stimulated of the neutrophils in the microchannel with 500 nM PMA. **(A)** Schematic illustration of the different steps shows that the TIME-on-Chip easily enables on-demand attachment and detachment of the components to initially induce the neutrophils in the microchannel to produce NETs, add the conditioned media to the spheroids, and re-attach the device to enable NETs-spheroid co-culture for analysis. **(B)** More than 60% of the neutrophils in the microchannel NETosed upon PMA stimulation for 6 h. But even after 24 h of co-culture, the spheroids do not appear significantly distorted as seen in the brightfield images (scale bars represent 150 μm). **(C)** No significant difference was observed in the distortion of the spheroids with the PMA-stimulated NETs present in the channel or with direct stimulation of the spheroids with PMA (data collected for *n* = 3 spheroids, mean ± SEM, *t*-test, ns, not significant). Reprinted from Surendran et al. (168), license CC BY 4.0.

Surendran et al. investigated how the tumor spheroids responded to neutrophil extracellular traps (NETs) produced in the microfluidic channel (168). They leveraged the advantage of magnetic hybrid integration technique for on-demand attachment and detachment of the ensembles, as schematically shown in Figure 9A. The neutrophils we cultured separately in the microfluidic channel and stimulated for NETs with 500 nM PMA for 6 h. The conditioned media that seeped out of the porous channel was collected and added back to the collagen layer on top of the spheroids and incubated for 1 h, before attaching the channel with the spheroid-collagen assembly. More than 60% of the neutrophils NETosed within the channel upon PMA stimulation (Figure 9B). The resultant spheroid distortion observed after 24 h was negligible. No significant difference was observed in the distortion of spheroids due to either direct stimulation with PMA or by stimulation of neutrophils in the channel with PMA (Figure 9C). Thus, the data presented clearly suggested that stromal NETs, and not the tumor-contacted NETs or the vascular NETs, play a significant role in mediating the collective invasion of cancer cells from an aggregated state.

**Figure 10:**
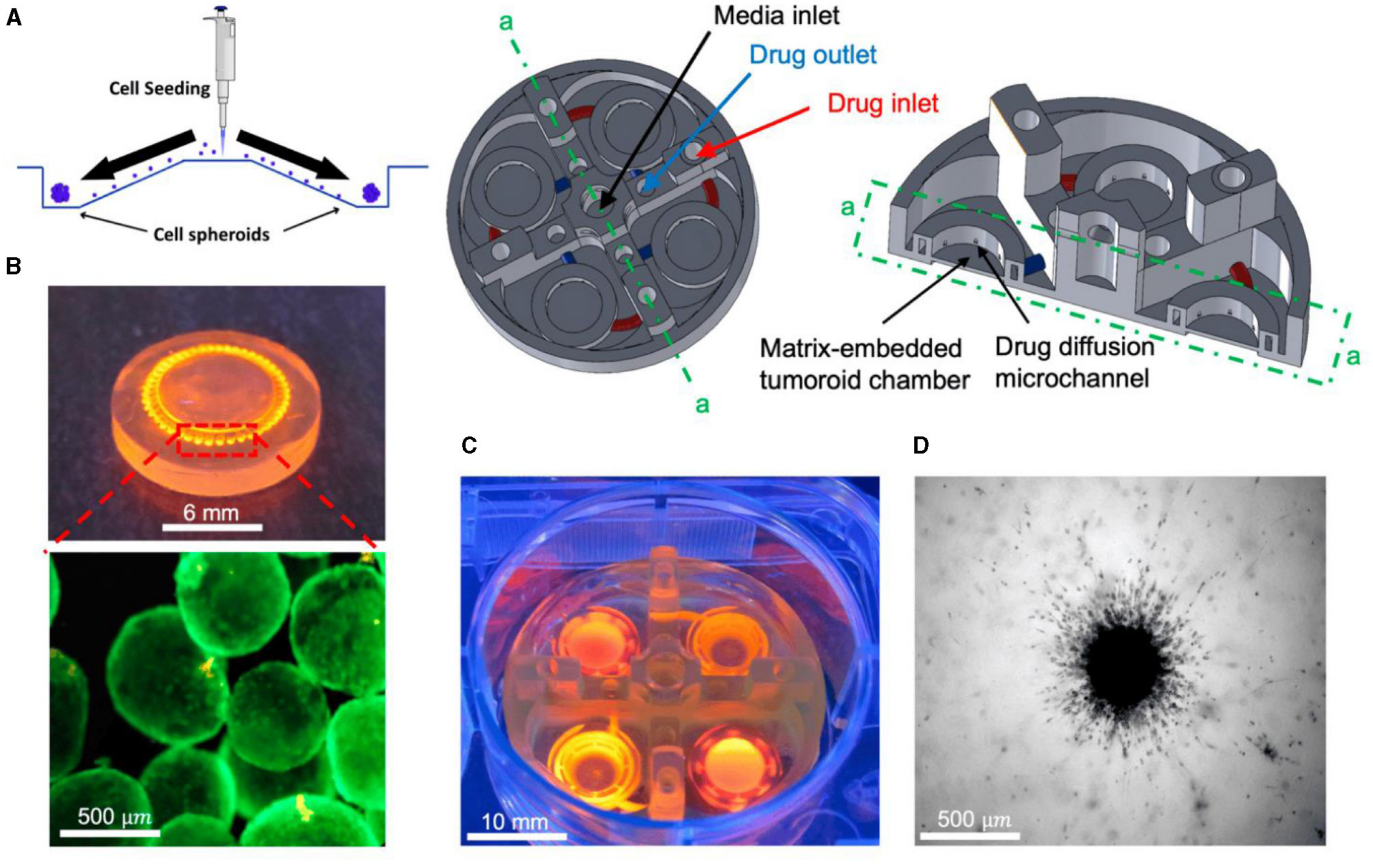
*In vitro* tumoroid invasion platform. **(A)** Single cell suspension seeded through the loading zone of a self-filling microwell array. **(B)** Tumoroids were formed after 4 days of culture and were transferred into the tumor-on-a-chip platform. **(C)** The platform was capable of growing tumoroids in four different chambers, each addressed separately, with an inlet and outlet for collagenase treatment. **(D)** Tumoroids embedded in bovine fibril collagen hydrogel were loaded into the open surface tumoroid-on-a-chip platform and their growth and invasion were monitored over time. Reprinted from Amereh et al. (169), license CC BY 4.0.

Amereh et al. investigated the influence of matrix stiffness on the growth and invasion of human glioblastoma tumoroids using a PEGDA-printed ToC platform (Figure 10) (169). Collagenase was used to create a heterogeneous collagen cellular matrix. The study allowed to observe a clear dependence of tumor behavior on changes in the rigidity of the extracellular matrix environment. The results showed that tumoroids exhibited higher growth rates and invasion lengths in response to higher concentrations of collagenase. These findings highlight the potential of investigating the impact of various matrix characteristics on tumor growth and invasion. Such investigations may unveil novel therapeutic targets and strategies for combatting glioblastoma's aggressive behavior in a more physiologically relevant context.

Dorrigiv et al. presented a hybrid modeling technique that combined a continuum reaction–diffusion model and a discrete model to accurately capture the growth and invasion in an inhomogeneous environment (170). The agreement between the experimental results and the model predictions further confirmed the validity and potential of this approach. Extending the ToC platform to incorporate other components of the tumor microenvironment could offer a more comprehensive representation of the complex tumor–stroma interactions. This advancement would enable the study of how different cellular and extracellular components contribute to glioblastoma progression in response to varying matrix characteristics (168–172).

### Sensorization of ToCs

The integration of the sensors with brain ToC platforms to provide real time spatiotemporal information of the tumor microenvironment provides critical insights of cancer progression and understanding of responses to cancer treatments (173). New generation ToC devices show typical characteristics of brain complexity, including the presence of different cell types, separation in different compartments, tissue-like three-dimensionality, and inclusion of the extracellular matrix components (45, 174). Moreover, the incorporation of a vascular system and mimicking the blood-brain barrier (BBB) makes brain ToC particularly attractive, since they can be exploited to test the brain delivery of different drugs and nanoformulations (174).

For real time monitoring of ToCs the best solution would be the integration of sensors into the device to detect physical parameters: temperature, pressure, density, conductivity, electrical impedance, magnetic field, absorbance, fluorescence, etc.; chemical parameters: pH, presence of particular compounds and drugs; biochemical parameters: target proteins, hormones, peptides, etc.; microphysiological parameters: speed of growth, etc.

**Figure 11:**
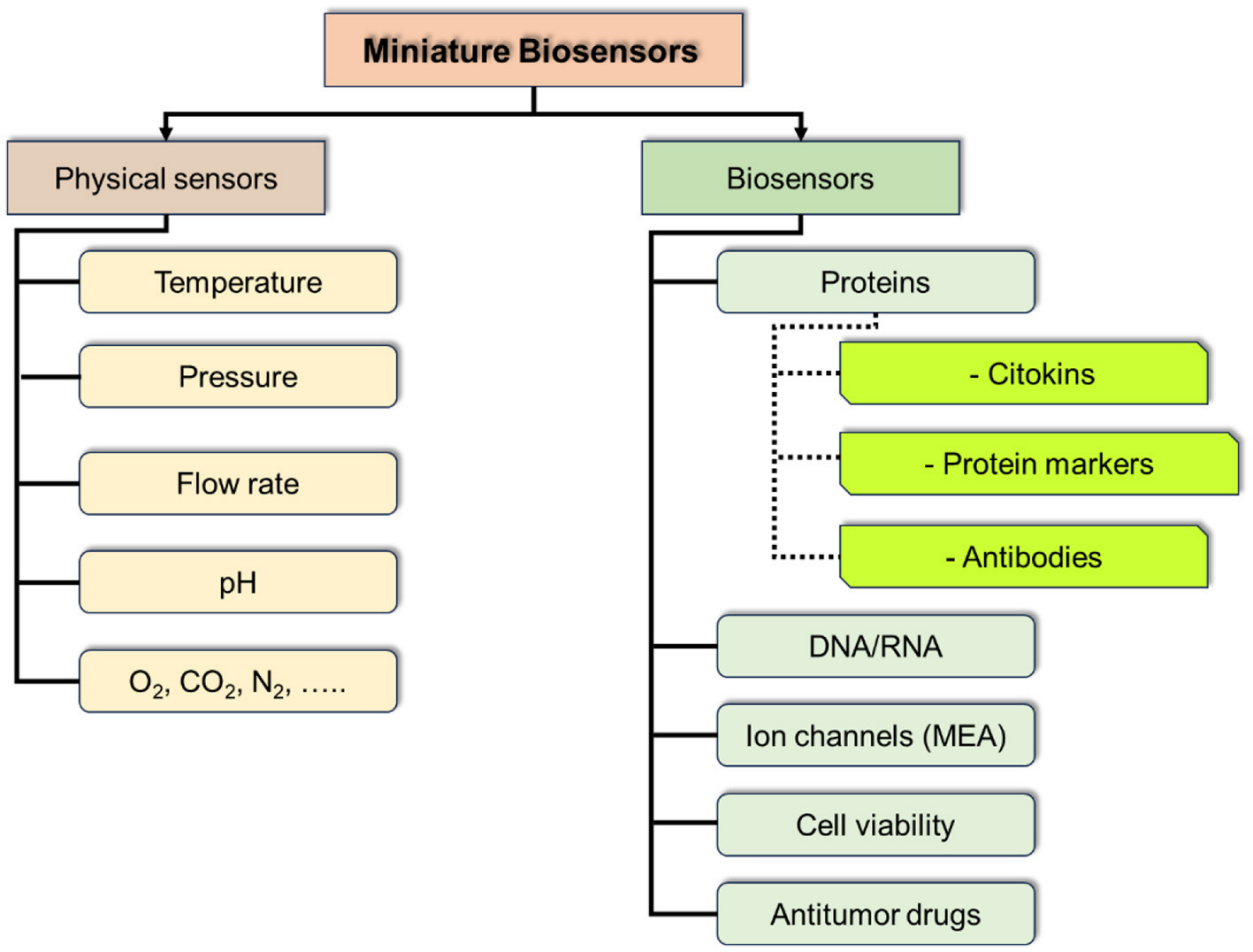
Schematic presentation of tissue types and indication of main sensor.

At the current stage of ToC development the integration of massive sensors could be of a particular interest and provide a breakthrough in studying tumor cultures and their environment, including the dynamics of the processes and response to physical and chemical interventions. Such sensor massive should include physical sensors for control of culture media parameters (temperature, pressure, pH, etc.), and chemical and biochemical parameters (marker proteins, antibodies, DNA/RNA, methabolites, etc.; Figure 11).

Among physical sensors it is necessary to mention recent achievements in fabrication of miniature planar (MEMS/NEMS) sensors integrable into the ToC systems. While developing microfluidic ToCs it is important to consider the group of sensors, which could help to mimicry the biosystem for culture growth by monitoring and adjusting the conditions of the medium (175). These parameters include pressure, particularly monitoring of narrow pressure ranges, using of a minute differential pressure sensor (MDPS), which have found applications in biomedicine, underscoring their significant engineering and medical value. An MDPS generally refers to a pressure sensor capable of measuring pressure ranges below 10 kPa (176, 177).

**Figure 12:**
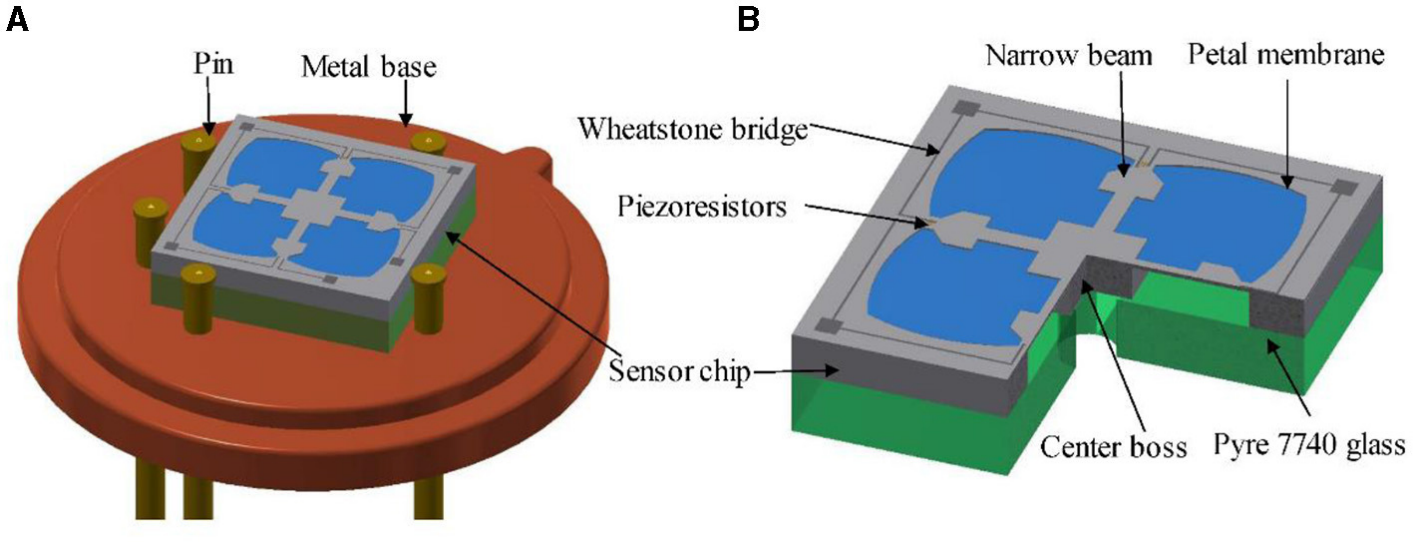
A diaphragm structure of a piezoresistive pressure sensor with a combination of a four-petal membrane, four narrow beams and a central boss (PMNBCB) for low-pressure ranges. General outlook **(A)**, and cross-section **(B)**. The finite element method (FEM) was used to estimate the stress distribution and analyze the inherent structure's deflection for different parameters. Reprinted from Tran et al. (178), license CC BY 4.0.

There is a growing demand for MDPSs with superior measurement resolution, enhanced frequency response, reduced size, and cost-effectiveness. To satisfy the sensitivity requirements of MDPSs, researchers introduced a variety of sensitive structures for ultrasensitive MEMS sensors (178–181), witnessing progressive enhancements in sensitivity (Figure 12).

Another important parameter is temperature of medium inside ToC. There is a number of solutions of cost effective, integrated sensors. One of approaches is using surface plasmon polaritons (SPPs), which are transverse electromagnetic (TM) waves that propagate along the metal-dielectric interface and decay exponentially away from the metal-dielectric juncture. SPPs have a swift and sensitive response to changes in the ambient medium, which opens new aspects in RI (refractive index) sensing applications. They can tune light-metal interactions and have a wide range of applications in sensors (182, 183) as well switches (184), and other light-on-chip systems useful for operation of life environment.

Another group of sensors essential for monitoring of tumor cells cultivation are the biosensors. A biosensor is defined as an analytical device that incorporates a biological sensing element connected to a transducer to convert an observed response into a measurable signal, whose magnitude is directly proportional to the concentration of a specific chemical/biochemical or a set of chemicals in the samples. Among biosensors, the most widely used are electrochemical and optical methods of signal detection.

Electrochemical transducers use electrochemical reactions to detect and measure biosensing element (bioreceptor) signals (185). Electrochemical biosensors usually use measurement principles such as amperometry, voltammetry, potentiometry, and impedimetry (186, 187). Electrochemical biosensors have advantages such as high sensitivity, specificity, low cost, compact size, integrability, and real-time monitoring capabilities. However, electrochemical biosensors also have disadvantages, such as the need for a reference electrode and electrolyte, as well as possible technological difficulties in immobilizing a biorecognition element into an organ-on-a-chip system. Using electrochemical biosensors, it is possible to monitor tumor parameters such as molecular biomarkers (proteins, nucleic acids), whole cells, cellular activity, metabolism, etc. (188–190).

**Figure 13:**
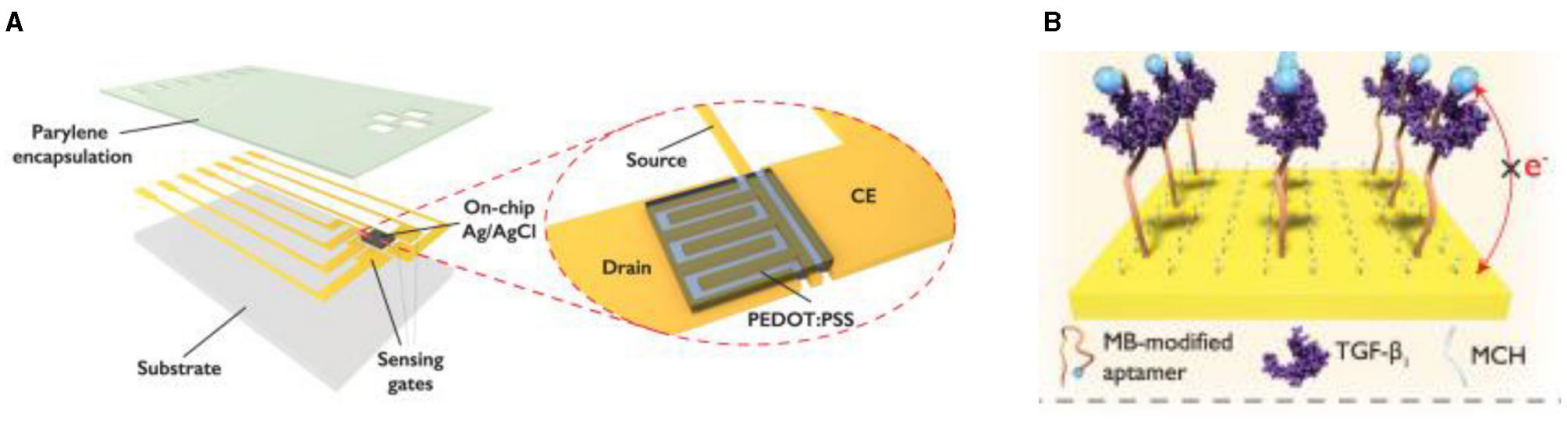
Design concept of ref-OECT-based E-AB sensor. **(A)** Schematic image of the ref-OECT-based E-AB sensor. **(B)** Sensing mechanism of the ref-OECT-based E-AB sensor for TGF-β1. Without the existence of TGF-β1, the methylene blue (MB) redox reporter is closer to the gate electrode surface, which results in a high gate current (IG) as well as a larger channel current modulation (IDS). In the presence of TGF-β1, a conformational change occurs in the aptamer, and the MB redox reporter moves further from the gate electrode surface, which results in low gate current and smaller channel current modulation. Reprinted from Ji et al. (191), license CC BY 4.0.

In some cases when a very high sensitivity of biosensors is necessary the authors propose a solution described by Ji et al. for implementation of electrochemical-aptamer based (E-AB) sensor incorporating organic FETs for amplification of the signal (ref-OECT-based E-AB sensor) produced by capture of Transforming growth factor beta 1 (TGF-β1) (191). This device can directly amplify the current from the electrochemical aptamer-based sensor via the in-plane current modulation in the counter electrode/transistor channel. The integrated sensor can sense TGF-β1 with 3–4 orders of magnitude enhancement in sensitivity compared to that in an electrochemical aptamer-based sensor (292 μA/dec vs. 85 nA/dec; Figure 13) (191).

A platform named “Digital Tissue-bArrier-CytoKine-counting-on-a-chip (DigiTACK),” proposed by Su et al. integrates digital immunosensors into a tissue chip system and demonstrates on-chip multiplexed, ultrasensitive, longitudinal cytokine secretion profiling of cultured brain endothelial barrier tissues (192). The integrated digital sensors utilize a novel beadless microwell format to perform an ultrafast “digital fingerprinting” of the analytes while achieving a low limit of detection (LoD) around 100–500 fg/mL for mouse MCP1 (CCL2), IL-6, and KC (CXCL1).

**Figure 14:**
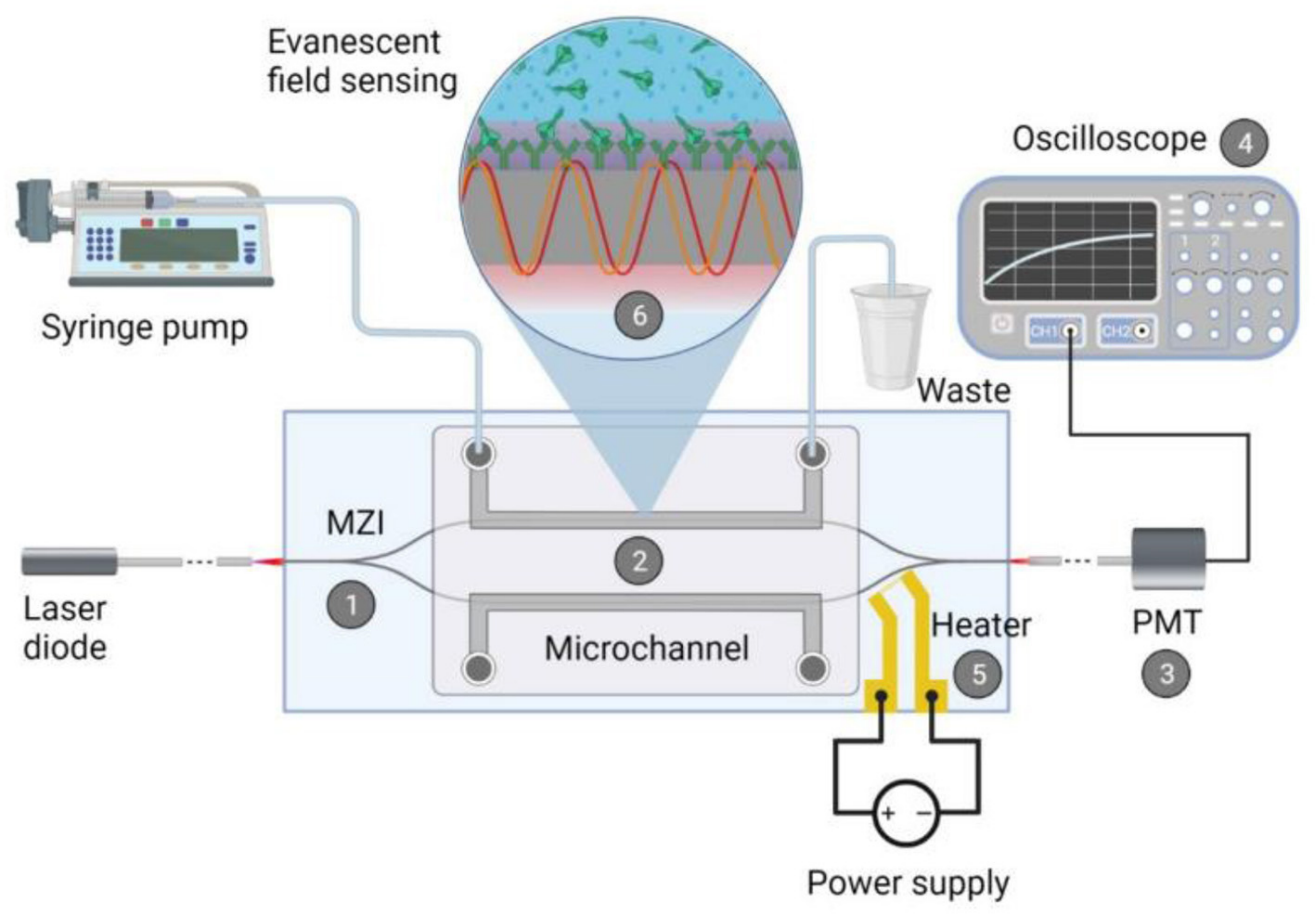
Schematic representation of the biosensor device: the integrated optical Mach–Zehnder interferometer (MZI) for sensing the analyte (1), the microfluidic apparatus (syringe pump, tubes, PDMS microchannel) for fluid sample providing (2), the signal processing unit, namely a photomultiplier tubes (PMT) detector (3) with an oscilloscope (4), the microheater structure for bias point tuning (5). The working principle of the device is also presented: the evanescent field detection is based on the phase difference in the propagating light of the measuring arm (yellow waves) compared to the ones of the reference arm (red waves) (6). Phase difference can be induced by the binding of the target spike protein S1 subunit to the antibody-covered surface of the measuring arm. Reprinted from Petrovszki et al. (196), license CC BY 4.0.

Optical biosensors use light radiation to detect and measure bioreceptor signals. Optical biosensors can be based on various principles, such as fluorescence, luminescence, absorption, scattering, interference, and others (193, 194). The key advantage of such biosensors is that there is no need for physical contact between the sensor element and the detector (195). In addition, they have high sensitivity, specificity, resolution, and dynamic range, and allow for multi-channel and multiplex analysis. Disadvantages of optical biosensors include the need for special light sources and detectors, the possible use of tags to increase sensitivity, and sensitivity to interference and contamination. Schematic representation of an integrated optical Mach-Zender interferometer (MZI) for the detection of target spike protein S1 subunit crossing the BBB after coronavirus infection (196) (Figure 14). As a light source a visible red laser diode (658 nm, 100 mW, RLT650-100MGS, Roithner Lasertechnik GmbH, Vienna, Austria) was used. The continuous flow of the samples was provided. The schematic representation of the experimental setup and the integrated optical MZI sensor can be seen in Figure 14.

Optical transducers together with various biorecognition elements permit the real-time monitoring of different biological phenomena and markers, such as the release of growth factors, the expression of specific receptors/biomarkers, the activation of immune cells, cell viability, cell-cell interactions, and BBB crossing of drugs and nanoparticles.

One of the important tools for studying cell barriers are transendothelial electrical resistance (TEER) sensors (197), which are designed to assess their integrity and functionality. Such sensors measure the electrical resistance created by cell layers between two electrodes. This method allows us to estimate the density of intercellular contacts and barrier permeability, which is useful for testing drugs and studying the effect of various factors on cell permeability over time. To measure TEER, multimeters or impedance measurement systems are used, which generate alternating current and measure the voltage created on the cell layer. Modern devices can be integrated with automatic systems for data analysis and real-time monitoring (198, 199).

Other types of sensors and biosensors in addition to the above-mentioned once can be integrated into ToCs for modeling and studying brain tumors. They include magnetic, mechanical, etc. (200–202). These sensors can work either alone or in combination with more common detection systems (203).

### Modeling vascularized tumor on a chip

Vascularized tumor on a chip (VToC) entails emulating intricate microvascular networks like those observed in tumors through microfluidic devices, which are meticulously designed to offer a faithful representation of cancer *in vitro*, exploration of tumor biology, evaluation of drug efficacy, and anticipation of patient responses to therapies (204). In comparison with traditional systems VToC demonstrate advantages by creating the environment in which physiological conditions for study of tumor-host interactions are of decisive importance for tumor development and for therapy sustainability.

As mentioned earlier, despite aggressive surgery, radiotherapy and chemotherapy, malignant gliomas remain uniformly fatal (56, 205). To progress, these tumors stimulate the formation of new blood vessels through processes driven primarily by vascular endothelial growth factor (VEGF) (206). But the vessels forms are appeared structurally and functionally abnormal, and are contributing to building of a hostile environment, including low oxygen pressure along with interstitial fluid pressure, which altogether lead to a more malignant phenotype with elevated morbidity and mortality (207). Emerging preclinical and clinical data indicate that anti-VEGF therapies are potentially effective in glioblastoma and can transiently normalize tumor vessels. This creates a window of opportunity for optimally combining chemotherapeutics and radiation.

The inhomogeneous density of tumor vessels causes anomalous blood flow. Local leaks may cause inhomogeneous circulation and consequently oxygen and drug delivery. This effect along with the other morphological anomalies of the system, lead to independence of the erythrocyte velocity on the vessels diameter, and is one-to-three orders of magnitude lower than it is observed in the normal pial vessels in gliomas and mammary carcinomas growing in the mouse brain (208). The velocity of erythrocytes movement cannot be measured in patients, while the blood flow velocity measured using functional MRI in patients with malignant gliomas, is abnormally being increased.

While MRI is typically being used for MPI for patients with brain tumor monitoring, a new method of vascular MRI is used for monitoring of structure and function of vessels in these patients.

Angiogenesis contributes to the progression of various malignant tumors. The primary cause of cancer death results from metastasis, which causes up to 90% of cancer-related mortality (209). Metastasis—a multistep process—is the migration and spread of cancer cells from the initial tumor to distant organs via vessels as well as their uncontrolled growth (210, 211). In the metastasis process, both collaborative and antagonistic interactions between host microenvironmental factors and tumor cells take place (212).

Limited blood supply and rapid tumor metabolism within solid tumors leads to nutrient starvation, waste product accumulation and the generation of pH gradients across the tumor mass. These environmental conditions modify multiple cellular functions, including metabolism, proliferation, and drug response (213). Thus, tumor cells located nearby blood vessels have enough nutrients to keep growing, forming a proliferative outer perimeter. Conversely, those cells located in the innermost region die of nutrient starvation, generating a necrotic core in the center of the tumor (214). Analysis of multiphoton images from tumor and normal tissue showed an average blood vessel diameter of 9.50 ± 0.04 μm for tumor tissue and 6.95 ± 0.36 μm for normal contralateral cortex tissue (214).

**Figure 15:**
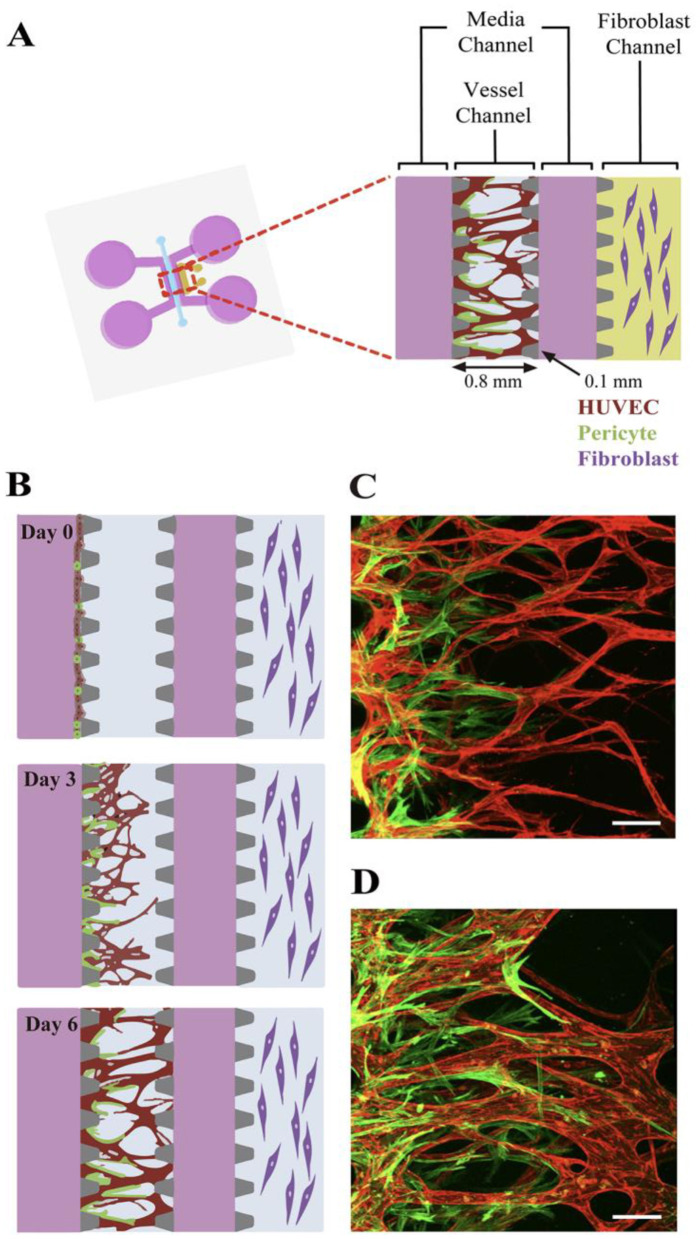
Schematic of the microfluidic system used to mimic the stepwise endothelial-pericyte interaction. **(A)** The microfluidic device is composed of a central vessel channel, two adjoining media channels, and the outermost fibroblast channel. The vascular network covered by the pericytes was formed in the central channel with assistance from the lateral fibroblasts. **(B)** The experimental scheme of the stepwise angiogenic process. ECs and pericytes were mixed and attached to the left side of the vessel channel. ECs sprout through the fibrin gel to establish a blood vessel network, and pericytes follow behind the vessel. **(C, D)** Confocal images show EC patterning prior to pericyte association during the first 3 days **(C)**, and matured pericytes covered the perfusable EC network on day 6 **(D)**. Scale bars, 100 μm. Reprinted from Kim et al. (216), license CC BY 4.0.

The described models recapitulate and systematically simplify the *in vitro* tumor microenvironment. This enables the study of a metastatic process in unprecedented detail. In modeling angiogenesis various approaches maybe perspective, such as: vascularization, microfabrication, bioprinting, etc. The attempts are made to culture the vascular system in the tumor model via using natural cell diversity. Thus, pericytes are cells present at intervals along the walls of capillaries (and post-capillary venules). In the CNS, they are responsible for blood vessel formation, maintenance of the blood–brain barrier, regulation of immune cell entry to the central nervous system (CNS) and control of brain blood flow (215). A method of development angiogenesis process, a so-called neovascularization (NV) on a microfluidic platform using pericytes is described in Kim et al. (216). NV is a multistep process including initial vascular angiogenic sprouting, followed by migration and association with pericytes and smooth muscle cells (217). Kim et al. engineered physiologically relevant *in vitro* vascular networks recapitulating the physical interaction between endothelial cells (ECs) and pericyte, as well as the development process of neovascularization (216). They demonstrated that the microfluidic model of angiogenesis can be used as a reliable experimental platform to form a perfusable vessel network derived from the co-culture of multiple cell types (Figure 15).

**Figure 16:**
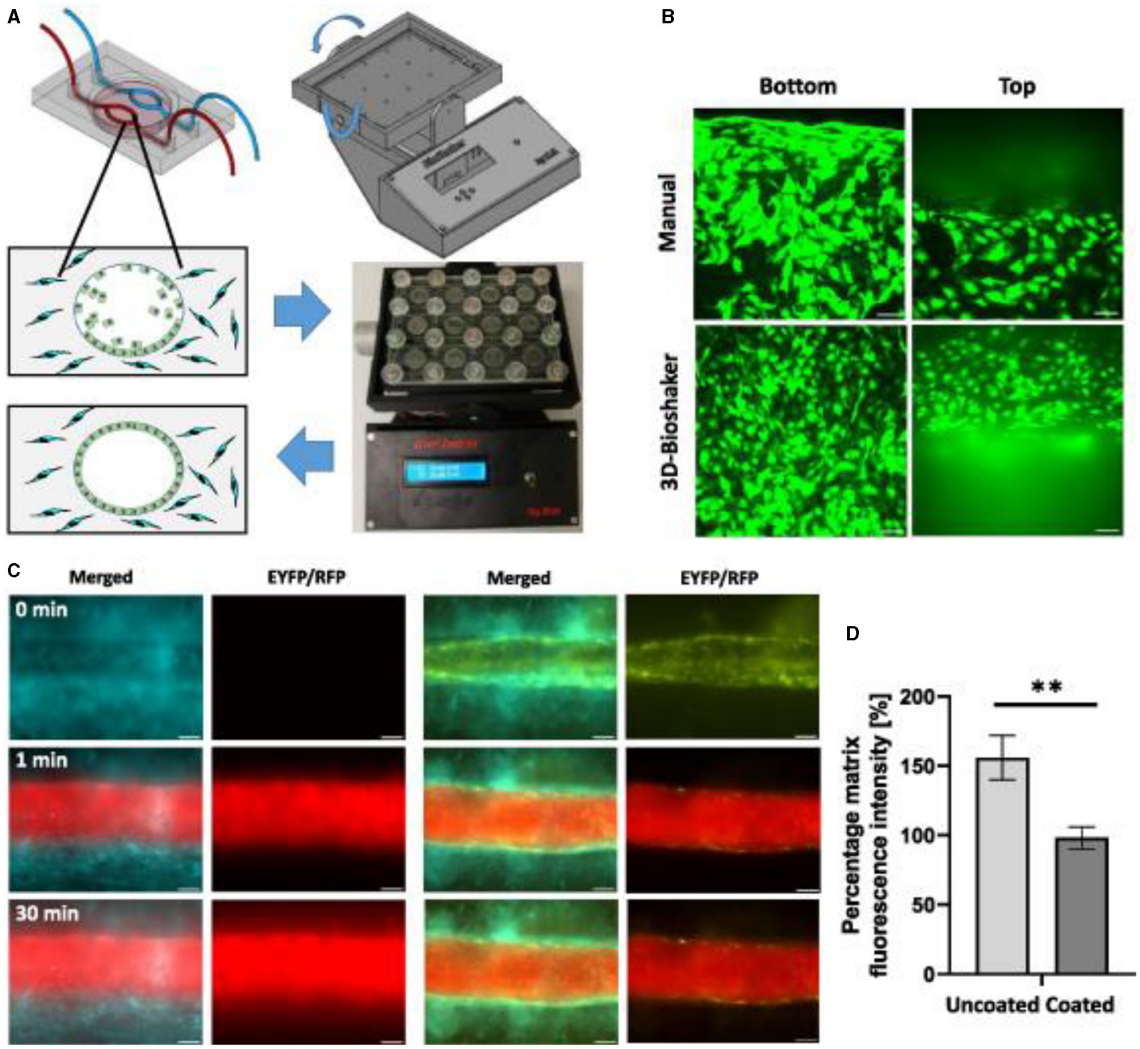
Endothelial cell coating of conduits to mimic blood-tissue barrier function. For optimized coating of inner channel linings, a programmable 3D orbital shaker was developed that halts rotation at distinct positions for defined times to achieve optimized endothelial cell adhesion **(A)**. Compared to manual flipping of the fluidic chips [**(B)**, top panels], the inner surfaces of shaker-incubated tissues are homogenously coated with green fluorescent endothelial cells (HUVEC/hTERT-EYFP) as imaged by confocal microscopy [**(B)**, bottom panels, scale bar 50 μm]. Tissue chips with fibroblasts (HFF/hTERT-ECFP) in the hydrogel matrix and hollow channels with or without endothelial cells (HUVEC/hTERT-EYFP) were perfused with red fluorescent 70 kDa dextran rhodamine B conjugate (0.25 mg ml^−1^) to assess diffusion into the hydrogel matrix within 30 min **(C)**. Left panels: fibroblast containing hydrogels with uncoated channels. Right panels: endothelial cell coating using orbital shaker procedure. Size marker 200 μm. Red fluorescence in the matrix after 30 min corresponds to the diffusion distance of dextran beads and thus to the endothelial barrier function. Graph shows comparison in red fluorescence between non-coated channels and channels covered with endothelial cells **(D)**. Shown is the mean of three independent experiments. Reprinted from Nothdurfter et al. (217), license CC BY 4.0. Statistical significance was assessed with student's t-test (^**^p < 0.01).

Thus, the microfluidic platform enabled to mimic the *in vivo* neovessel formation followed by pericyte coverage. The microfluidic device was composed of a central vessel channel for engineering pericyte-covered microvessels *in vitro*, two adjoining media channels, and the outermost fibroblast channel (Figure 16A). A mixture of ECs and pericytes was attached to one side of the central a cellular fibrin matrix. In response to the directional gradient of biochemical factors secreted by fibroblasts, leading cells of EC sprouts grew toward the opposite end of the central channel and spontaneously formed vacuoles that merged into the intracellular lumen (Figures 16B, C). After 5 or 6 days of culture, when the leading portion of the angiogenic sprouts reached the end of the fibrin matrix, ECs robustly developed interconnecting and perfusable vascular networks with tightly adhered pericytes on the basolateral surface of the blood vessels (Figures 16B, D) (217). Matrix based on gelatin-methacrylate/fibrin was combined with endothelial cells to imitate the microenvironment of the tumor. It has been demonstrated that adipocyte- and induced pluripotent stem cells-derived mesenchymal stem cells can manage angiogenesis.

Bioprinted channels are coated with ECs post printing to form a dense vessel-tissue barrier. Patient-derived neuroblastoma spheroids are added to the matrix during the printing process and grown for more than 2 weeks. Presented the first bioprinted, micro-vascularized neuroblastoma—tumor-environment model directly printed into fluidic chips and a novel medium-throughput biofabrication platform suitable for studying tumor angiogenesis (Figure 16).

The development of new methods and technologies for modeling brain tumors is essential for improving the accuracy of their vital functions research and developing new therapeutic approaches. Traditional 2D tumor models are unable to fully reproduce the complex architecture and life cycles of tumors, making it difficult to predict their behavior and response to treatment. The use of microfluidic chips in combination with 3D cultivation methods allows for a more accurate recreation of the natural conditions of the tumor environment. 3D cultures, such as spheroids and organoids (218), more accurately reflect the interaction of cells and their behavior, while preserving their natural characteristics. This improves the reproducibility and relevance of experiments and allows for the study of tumor cell growth, metastasis, and invasion processes. In addition, the use of ToC systems opens up new opportunities for studying the tumor microenvironment and the dynamics of their development under conditions close to physiological ones. Despite the progress, difficulties remain, such as the high cost and complexity of reproducing tumor structures. In the future, further developments in the field of micro- and nanotechnology, bioprinting, and organoid technologies can significantly expand the possibilities of testing antitumor drugs and studying their impact on various stages of tumor growth.

### Modeling of blood-brain barrier on a chip

Techniques of BBB modeling *in vitro* date back to 1953, when the original monolayer cell culture transwell system (developed by Corning Inc., Corning, NY) was utilized (219, 220). The late 2000's and early 2010's saw the emergence of dynamic and 3D models such as organoids or barriers on chips using microfluidics (221). Now there is many *in vitro* methods available that can be used in drug development, disease modeling, neurotoxicity screening, and personalized medicine applications to estimate the permeability of a substance across the BBB (222, 223). Drugs targeting the central nervous system (CNS) are 45% less likely to enter Phase III trials than non-CNS drugs, according to data from 1990 to 2012 (224).

The oldest and best-established method to study the transport of molecules across the BBB is to perform *in vivo* experiments, having their advantage since it allows for studies of the brain in its natural environment, at the same time such experiments are unethical (220). Since human studies are restricted to post-mortem investigations or imaging techniques such as magnetic resonance imaging (MRI) and positron emission tomography (PET) with limited resolution, most research on the BBB still involves laboratory animals (225). *In vitro* models are comparatively less complex, and flexible, as per the study design, could generate substantial evidence and help identify suitable *in vivo* animal model selection (226). All the modern types of *in vitro* BBB models have their specific advantages, limitations, and recommended areas of application (225). A useful BBB model should meet the following criteria: reproducibility of solute permeability, display of a restrictive paracellular pathway and physiologically realistic architecture, functional expression of transporters, and ease of culture (227). Some important challenges in the BBB models development include: (1) improved fidelity of the spatial arrangement of BBB cell types in a zonation-specific manner, (2) incorporation of blood components known to be important in communication between the vascular system and the brain, (3) developing strategies for multiscale/hierarchical models along cerebrovascular zones, and (4) recapitulating aspects of neurovascular coupling (228). The current BBB models use transwell culture inserts, microfluidic devices, and models based on cell aggregates consisting of a human bone marrow microvascular endothelial cells, astrocytes and pericytes (229).

BBB-on-chip models (microfluidic *in vitro* BBB models or μBBB) can potentially be used as preclinical drug screening and toxicological assessment tools and are being developed intensively (230, 231), similarly to the other tumor-on-a-chip models including vascularized structures providing cell culture and co-culture and also the cell-free models [e.g., parallel artificial membrane permeability assays (PAMPA)] (222, 232). Brain-on-a-chip models have been developed in an attempt to address many of the limitations found in other models and have been used with human cells and dynamic systems to create microphysiological models exhibiting specific functions and unique brain tissue regions (233). The design of a μBBB must consider two main areas: the “blood side,” mimicking the microvascular lumen of the brain capillary bed; and the “brain side,” mimicking the abluminal side of the capillaries and the brain parenchyma (234). μBBB exhibit several key advantages (235): the chips are easy to design and fabricate; microchannel sizes are similar to microvascular structures *in vivo*; multi-dimensional network structures resemble the microenvironment *in vivo*; it is straightforward to combine and integrate various functional units.

One of the common approaches in modeling BBB is to measure the electrical resistance across the barrier with a technique called transendothelial electrical resistance (TEER), which uses electrical resistance between two electrodes placed across the BBB (often used in combination with growing endothelial cells in a transwell model) as a surrogate measure of permeability (75). Since the study of TEER and permeability alone does not offer a great insight on pharmacophysiology of BBB due to its association with a complex communicating module, which makes it important to fabricate the whole cerebrovasculature on a chip (207). In this case, for example, a modular chip housing the 3D culturing of ECs from rat brain with 95% of astrocytes, 1% of microglia and rest of neuronal cells can serve as a potential tool for screening the therapeutic agents developed to cure neurological diseases (207).

*In vitro* BBB models include cell culture models, brain slice models, fiber-based dynamic *in vitro* BBB (DIV-BBB) models, and μBBB (235). Organoid-based BBB models have also been developed as cell-based, 3D models in the absence of biomaterials (236). Organoids are self-organized cellular structures that can be derived directly from patient tissue or using developmental biology and exhibit characteristics of several organs, which include brain, along with pancreas, gut, retina, and brain (237). The native patient tissue, instead of the standard cell lines, is used to make organoids-on-chip a real part of pre-clinical and clinical research in the field of effective precision medicine (237).

Despite the quite long period of BBB-models development, this area is still attracting scientific interest (238). The use of μBBB can improve BBB modeling by having more realistic dimensions and geometries, and by exposing the endothelium to physiological fluid flow (36). Some authors additionally introduce “μBBB+” models considering non-malignant disorders (239). Developmental and degenerative brain diseases can be characterized with pathological biomarkers often associated with cerebral flow and vasculature (240). The microfluidic BBB model can provide opportunities to regulate the transport of signaling molecules, nutrients, growth factors, and drugs for biological research without incorporating animal or human models, limiting ethical concerns (241). Fluid flow at the BBB plays an important role in maintaining barrier functions and homeostasis of CNS, which is partially caused by shear stress generated by circulatory blood flow in the brain capillaries ranging from 5 to 23 dyn/cm^2^ (242). The currently existing systems for creating flow inside the microfluidic chip can be divided into the two main groups: passive (including gravity-induced, capillary action, surface tension, vacuum suction, osmosis, pressure-driven) and active (syringe pumps, micropumps, electromagnetic and magnetic valves, and vacuum pumps). One of the most commonly applied methods to create flow in microfluidic devices is a use of syringe pumps (243).

One of the most common used platforms of *in vitro* BBB models is created using transwells with 10 μm thick polystyrene or polycarbonate membranes with 400 nm pores with a 10^8^ pores/mm^2^ pore density (220). This BBB model involves the growth of a monolayer of endothelial cells in a transwell insert, which represents the blood or the luminal side, whereas the well in which it is inserted represents the abluminal or parenchymal side (244). This quite simple BBB model is still intensively used and most capable of high-throughput drug screening due to its low cost and relatively easy use (245, 246). The neurovascular unit (NVU) is the minimal functional unit of the brain. It is composed of vascular cells, glial cells, and neurons (247). BBB models can employ primary and immortalized cells from human, rodent, bovine, and porcine sources, neurovascular unit (NVU) cells derived from pluripotent stem cell or neural stem cell sources.

NVU is the system responsible for maintaining the proper internal environment of the CNS through control and modulation of BBB functions and cerebral blood flow (248). The crosstalk between the cells of the NVU is crucial for the formation and maintenance of a functional BBB (249). Up to date, one of the most complex models of the BBB is NVU-on-a-chip (248). Modulation of stem cells differentiation into BBB phenotypic cells can boost the development of clinically relevant NVU models for basic and translational studies and drug development (249). Stem cells could deliver a breakthrough in BBB modeling allowing for the development of the desired cell cultures *in situ* within the platform itself (250).

**Figure 17:**
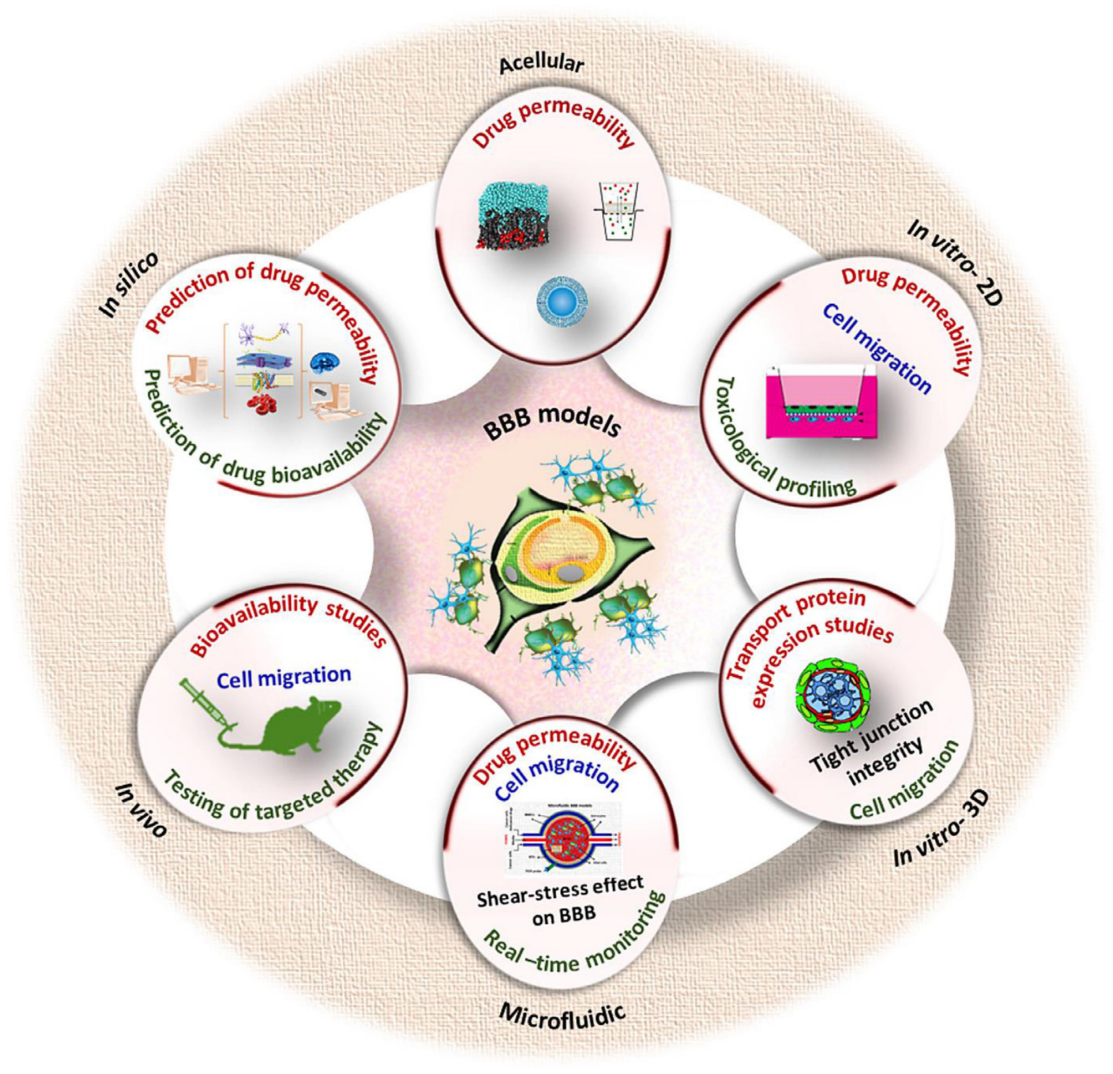
A scheme showing the application of various BBB models in drug development and pharmaceutical research. Reprinted from Augustine et al. (251), license CC BY 4.0.

A brief outline of BBB models and their major applications in drug development and pharmaceutical research is provided in Figure 17 (251).

Models comprising pluripotent stem cells-epithelial cells and primary astrocytes/glioma cells, pericytes (and neurons), were revealed to be the most suitable replacements for current *in vivo* models for the purpose of assessing permeability (252). 3D methods have been evolved into emergent 3D systems, such as self-assembled microvasculatures via angiogenic sprouting or vasculogenesis and organoids that enable the generation of all brain cell types, in some cases with internal vasculatures (253). The inclusion of human-derived cells, tissues, and patient samples is highly recommended to improve predictive power not only in cancer, but also in non-malignant disorders, including dementia research (254). The development of *in vitro* 3D models able to emulate the intrinsic features of the NVU in a concrete pathology may be of significant benefit to assess the transport of nanomedicines across the diseased cerebral vasculature and opens new avenues to assess the therapeutic potential of nanomedicines for these pathologies (255). 3D spheroid models are being increasingly developed for a variety of pediatric brain cancers replicating elements of the tumor microenvironment such as a gradient distribution of nutrients, oxygen, pH, cell-cell, and cell-extracellular contact (255). Despite altered BBB functions are observed in several diseases of the CNS, little is known about possible tissue size-dependent effects on barrier function, which could severely limit the reproducibility of current *in vitro* spheroid models (256).

3D μBBB model can be successfully used to study the effect of physiological flow on BBB integrity, including the role of separated flow in the development of pathologies, such as atherosclerosis and aneurysm (257). A microfluidic system can be used for mimicking cellular blood vessel barriers providing means to study the transport of biomolecules (e.g., lipoprotein particles, extracellular vesicles, but also lipids and hormones) across the barrier (257). 3D μBBB models were shown to be perceptive in parasitology research and therefore can be applied to study various microbial agents with neurotropism (258). The utility of the human NVU chip was shown for the real-time observation and quantification of fungal brain penetration and neurotropism (259). Duong et al. (260) designed and fabricated a microfluidic device based on a sandwiched cellulose fiber membrane, which can be used as an easy and cheap functional 3D *in vitro* BBB model for short experiments. A conventional engineered transwell BBB model is still being actively used, and Vakilian et al. (261) demonstrated the anti-metastatic effect of β-boswellic acid by deteriorating cancer cells as well as improving barrier integrity. A millifluidic device compatible with standard transwell inserts was developed to examine the impact of primary human pericytes and astrocytes on human brain microvascular endothelial cells barrier integrity (262).

A synergistic engineering approach toward 3D vascularized glioblastoma multiforme (GBM)-on-chip model has been developed based on sealed microfluidic channels, endothelialized to mimic the BBB, together with a 3D bioprinted GBM model (263). The complicated GBM microenvironment and the anatomical features and functionality of the *in vivo* BBB was developed and the influence of the GBM microenvironment on tumor behavior and drug delivery was demonstrated with the use of a human BBB model based on co-culturing BBB-composing cells within a 3D hydrogel matrix (264). Studies with a use of 3D bioprinting and biomaterials to construct GBM and BBB models have demonstrated physiologically relevant properties and improved features compared to traditional models (265). The capability of the BBB-GBM platform was shown to screen therapeutic nanoparticles (NPs) and predict *in vivo* efficacy, demonstrating improved efficacy of cisplatin when encapsulated in GBM-targeting layer-by-layer NPs both *in vitro* and *in vivo* (266).

Preliminary data was shown to demonstrate how system based on co-culturing of tumor cells in a hydrogel mimicking tumor extracellular matrix along with a cellular barrier can be used to monitor the growth of micro-tumors and their interaction with a cellular barrier and migration into the perfusion channel (267). Hajal et al. (268) fabricated an engineered human μBBB model and measured physiologically relevant molecular permeability, which may be used in academic or industry laboratories to study and predict transport across the BBB. The impact of shear stress was demonstrated to an easy to isolate, user-friendly and highly reproducible BBB model derived from human brain cell line and human primary astrocytes co-cultured under continuous laminar flow which can be used to study the permeation and migration of different cell types through a tightly formed BBB under healthy and inflammatory states (269). In the single- and double-channel devices, Martins et al. (270) developed a 3D GBM model and simulated the direct injection/application of chemotherapy at the tumor site (i.e., *in situ*); the double-channel microfluidic chip has two parallel channels connected by micropillars to mimic the vascular and parenchymal-cancer compartment and therefor to simulate the systemic administration of chemotherapy.

Lam et al. (271) developed a model of GBM and its accompanying BBB that recapitulates clinically relevant features, such as tumor spatial heterogeneity and GBM-BBB interactions. The model allowed to investigate the role of tumor spatial heterogeneity and the BBB in Temozolomide resistance, and to carry out unlabeled proteomic screening to identify potential proteins involved in tumorigenesis and Temozolomide resistance. A tissue-engineered model was developed that provides new insight into changes in brain-tumor barrier phenotype during metastatic breast cancer, which may motivate new therapeutic approaches (228).

The effects of tumor treating fields (by example of electromagnetic field with a frequency of 100 kHz) were examined on the BBB in a 3D co-culture model consisting of primary human brain microvascular endothelial cells and immortalized human pericytes, temporary increase of BBB permeability in an *in vitro* model of human origin was proven (272). A BBB-glioma chip model was developed reconstituted with human brain microvascular endothelial cells, astrocytes, and pericytes under dynamic culture and U251 cells culturing in Matrigel as cells cluster to mimic the glioma microenvironment for the evaluation of permeability and drug efficacy of potential anti-glioma components of traditional Chinese medicine (273). A blood–brain niche microfluidic chip was developed to characterize alterations to the brain niche and cancer cell metastatic progression to characterize phenotypic and secretory cues provided by individual cellular residents of the brain niche, strocytes and microglia, which attract metastatic cancer cells (274).

The information summarizing various examples of up-to-date *in vitro* BBB models for neuro-oncology of the most popular 3D-cell culture microfluidic dynamic and transwell-based static types is given in Table 1.

**Table 1:** Some examples of modern *in vitro* BBB models for neuro-oncology.

**BBB model's type**	**Model's structure description**	**Cell types used**	**Potential applications**	**References**
3D-cell culture microfluidic dynamic model	Microfluidic chip with integrated multi-size spheroid array	Caco-2, normal human dermal fibroblasts, HepG2, A549, human primary ACs^a^ and PCs^b^, and human cerebral microvascular ECs^c^	Investigation of active and passive transport across the BBB	(256)
	Two fluidic regions flanking the hydrogel region to hold cell culture media	U87-ZsGreen cells, human brain microvascular ECs, primary brain PCs, and human ACs	Modeling of tumor's physical and cellular microenvironment	(271)
	A hydrogel matrix containing tumor cells or spheroids formed around a template rod	Induced pluripotent SCs^d^, brain microvascular endothelial-like cells, and human JIMT-1-BR cancer cells	Study of changes in BTB phenotype during metastatic breast cancer	(228)
	Two parallel channels connected by micropillars mimicking the vascular and parenchymal-cancer compartment	Human GBM^e^ (U87-MG) cells, human cortical ACs	Simulation of the systemic administration of chemotherapy	(259)
	A parallelizable microfluidic platform with 10 independent cell culture chambers	HT29 human colon adenocarcinoma cells, Madin-Darby canine kidney cells	Monitoring the growth of micro-tumors and their interaction with a cellular barrier	(267)
	Co-culturing BBB-composing cells within a 3D hydrogel matrix injected in a PDMS^f^ chip	Human brain microvascular ECs, human GBM T98G and U87MG cells	Replication of GBM microenvironment and the BBB anatomical features and functionality	(264)
	A chip with four channels separately contains human ECs, ACs, PCs and hydrogels, U251 and Matrigel, and culture medium	Primary human brain microvascular ECs, primary human ACs, primary human brain vascular PCs, and U251 glioma cells	Evaluation of permeability and efficacy of potential anti-glioma drugs	(273)
	Endothelialized microfluidic channels with a 3D bioprinted GBM model	Human glioblastoma cells A-172, human umbilical vein ECs, cerebral micro-vessel ECs	Study of the GBM progression under simulated microgravity condition	(263)
	GBM spheroid co-cultured with PCs embedded in a BBB vascular system	Human induced pluripotent SCs-derived ECs, human brain PCs and ACs, the high-grade GBM22 cells	Understanding of tumor-blood-vessel biology and the development of brain-penetrant therapeutics	(266)
	The SynBBB commercial chip with two microchannels and one micro-brain-chamber	Human umbilical vein ECs and human ACs	Study of a selective function of β-Boswellic Acid in the prevention of brain metastasis	(261)
	A chip with four blood-brain niche channels with a collagen solution-formed bottom chamber	Human microglia line HMC3, human ACs, human brain microvascular ECs, breast cancer JIMT-1 and JIMT-1-BR cells	Study of alterations to the brain niche and cancer cell metastatic progression	(274)
	Three interconnected main channels with microchannels fabricated using PDMS	Human brain microvascular ECs, ACs, U87MG GBM cells	The study of cell migration, tumor invasion, and organoid growth	(276)
Transwell-based static model	Transwell plate coated with collogen solution and seeded by cells	Human brain ECs, U87 and GL261 GBM cells	Efficacy improvement of Temozolomide in glioblastoma therapy	(275)
	3D co-culture model in transwell inserts with of 0.4 μm pores	Human brain microvascular ECs, immortalized PCs	Study of the effects of tumor treating fields on the BBB model	(272)
	Cell insert membranes coated with diluted Geltrex™ solution	Human umbilical vein ECs and human ACs	Study of a selective function of β-Boswellic acid in the prevention of brain metastasis	(261)
	Insert with a 3 μm pore size polyester membrane coated with collagen-I	Immortalized human brain microvascular ECs	Improved delivery of hydrophobic anti-cancer drugs to GBM	(277)

^a^Astrocytes.

^b^Pericytes.

^c^Endothelial cells.

^d^Stem cells.

^e^Glioblastoma multiforme.

^f^Polydimethylsiloxane.

Summarizing the information on the current state of the *in vitro* BBB models intended for perspective applications in the field of preclinical study of neuro-oncology drug testing, some main tendencies can be formulated. First refers to the use of the modern techniques including 3D dynamic models based on cell co-culturing in gel matrices. And the second refers to the continuous use of rather simple Transwell-based models despite of the more than 50 years history of their development and application. New techniques for modeling brain tumors on microfluidic platforms represent an important step in overcoming the limitations of traditional approaches and help accelerate the development of more effective and safe treatment.

## Conclusions

Tumor-on-a-chip (ToC) models play an important role in studying tumor microenvironment, invasion, angiogenesis, and drug response. Classification of such models is based on the type of simulated structure, the number of microchannels, and the presence of a vascular network, which allows the creation of both simple 2D models and more complex 3D systems to mimic developed vascular networks or i tumor behavior when interacting with the blood-brain barrier. Analyzing various approaches to creating ToCs, we can conclude that the creation of a specific type of system and the integration of various sensor elements into it is determined by the set of parameters that researchers need to obtain using such a system. Now, there is no optimal universal solution that would satisfy all the requirements and research goals. However, some general trends and directions in the development of ToCs can be identified that can improve their performance, functionality, and applicability.

There is a trend toward increasing the complexity and realism of models by reproducing more accurate and complete structure and function of the tumor microenvironment, including various types and ratios of cells, extracellular matrix, vascularization, barriers, gradients, forces, and other factors that influence the behavior and fate of tumor cells. Hybrid integration of modern micro- and nanotechnologies with cellular technologies opens new possibilities for creating high-precision models of brain ToCs. The use of microfluidics and microfabrication can more accurately reproduce the processes of angiogenesis, invasion, and tumor response to therapy. However, as the complexity of such systems increases, their cost also increases. This is due to the need for expensive equipment, complex materials, and high requirements for personnel qualifications. To make tumor-on-a-chip technologies more accessible, modular platforms can be introduced that will allow researchers to adapt the systems to their needs without significant costs. Active partnerships between academia and industry can reduce the cost of developing and distributing such systems. This will also allow the development of standardized solutions that will be easily integrated into research worldwide, providing broad access to advanced technologies.

In addition, there is a need to increase the degree of automation and standardization of models using more universal and interoperable platforms, protocols, interfaces, and software. This will make it easier and faster to create, configure, manage, and analyze tumor cultures using ToCs. In addition, the implementation of modern solutions based on information technology and artificial intelligence will simplify the management of complex experiments and the analysis of large volumes of data, which will make the research process not only faster but also cheaper. This will also lead to more efficient use of resources and a reduction in errors associated with manual operations. As a result, increasing the level of automation and standardization will be an important step toward the development of more accessible and scalable models in biomedical research. A separate challenge is to improve the sensitivity, resolution, and integration of models using more advanced and miniaturized sensors that can detect and measure a wider range of parameters related to brain tumors or their treatment, as well as transmit and process these signals in real time and with high accuracy and reliability.

Thus, ToCs for brain tumor mimicking represent a promising and innovative technology that can significantly improve the understanding of the mechanisms and processes associated with brain tumors, as well as contribute to the development of new and effective strategies for the early diagnostics, therapy, and prevention of this diseases.
